# UHRF1 ubiquitin ligase activity supports the maintenance of low-density CpG methylation

**DOI:** 10.1093/nar/gkae1105

**Published:** 2024-11-28

**Authors:** Rochelle L Tiedemann, Joel Hrit, Qian Du, Ashley K Wiseman, Hope E Eden, Bradley M Dickson, Xiangqian Kong, Alison A Chomiak, Robert M Vaughan, Bailey M Tibben, Jakob M Hebert, Yael David, Wanding Zhou, Stephen B Baylin, Peter A Jones, Susan J Clark, Scott B Rothbart

**Affiliations:** Department of Epigenetics, Van Andel Institute, 333 Bostwick Ave NE, Grand Rapids, MI 49503, USA; Department of Epigenetics, Van Andel Institute, 333 Bostwick Ave NE, Grand Rapids, MI 49503, USA; Epigenetics Research Program, Garvan Institute of Medical Research, 384 Victoria St, Darlinghurst, NSW 2010, Australia; Department of Epigenetics, Van Andel Institute, 333 Bostwick Ave NE, Grand Rapids, MI 49503, USA; Department of Epigenetics, Van Andel Institute, 333 Bostwick Ave NE, Grand Rapids, MI 49503, USA; Department of Epigenetics, Van Andel Institute, 333 Bostwick Ave NE, Grand Rapids, MI 49503, USA; Sidney Kimmel Comprehensive Cancer Center, The Johns Hopkins University School of Medicine, 401 N Broadway, Baltimore, MD, USA; Department of Epigenetics, Van Andel Institute, 333 Bostwick Ave NE, Grand Rapids, MI 49503, USA; Department of Epigenetics, Van Andel Institute, 333 Bostwick Ave NE, Grand Rapids, MI 49503, USA; Department of Epigenetics, Van Andel Institute, 333 Bostwick Ave NE, Grand Rapids, MI 49503, USA; Chemical Biology Program, Memorial Sloan Kettering Cancer Center, 1275 York Ave, NY, NY 10065, USA; Chemical Biology Program, Memorial Sloan Kettering Cancer Center, 1275 York Ave, NY, NY 10065, USA; Center for Computational and Genomic Medicine, Children's Hospital of Philadelphia, 3501 Civic Center Blvd, Philadelphia, PA19104, USA; Sidney Kimmel Comprehensive Cancer Center, The Johns Hopkins University School of Medicine, 401 N Broadway, Baltimore, MD, USA; Department of Epigenetics, Van Andel Institute, 333 Bostwick Ave NE, Grand Rapids, MI 49503, USA; Epigenetics Research Program, Garvan Institute of Medical Research, 384 Victoria St, Darlinghurst, NSW 2010, Australia; St. Vincent's Clinical School, University of New South Wales, 390 Victoria Street, Darlinghurst, NSW 2010, Australia; Department of Epigenetics, Van Andel Institute, 333 Bostwick Ave NE, Grand Rapids, MI 49503, USA

## Abstract

The RING E3 ubiquitin ligase UHRF1 is an established cofactor for DNA methylation inheritance. The model posits that nucleosomal engagement through histone and DNA interactions directs UHRF1 ubiquitin ligase activity toward lysines on histone H3 tails, creating binding sites for DNMT1 through ubiquitin interacting motifs (UIM1 and UIM2). However, the extent to which DNMT1 relies on ubiquitin signaling through UHRF1 in support of DNA methylation maintenance remains unclear. Here, with integrative epigenomic and biochemical analyses, we reveal that DNA methylation maintenance at low-density cytosine-guanine dinucleotides (CpGs) is particularly vulnerable to disruption of UHRF1 ubiquitin ligase activity and DNMT1 ubiquitin reading activity through UIM1. Hypomethylation of low-density CpGs in this manner induces formation of partially methylated domains (PMDs), a methylation signature observed across human cancers. In contrast, UIM2 disruption completely abolishes the DNA methylation maintenance function of DNMT1 in a CpG density-independent manner. In the context of DNA methylation recovery following acute DNMT1 depletion, we further reveal a ‘bookmarking’ function for UHRF1 ubiquitin ligase activity in support of DNA re-methylation. Collectively, these studies show that DNMT1-dependent DNA methylation inheritance is a ubiquitin-regulated process that is partially reliant on UHRF1 and suggest a disrupted UHRF1-DNMT1 ubiquitin signaling axis contributes to PMD formation in cancers.

## Graphical Abstract

**Graphical Abstract:**
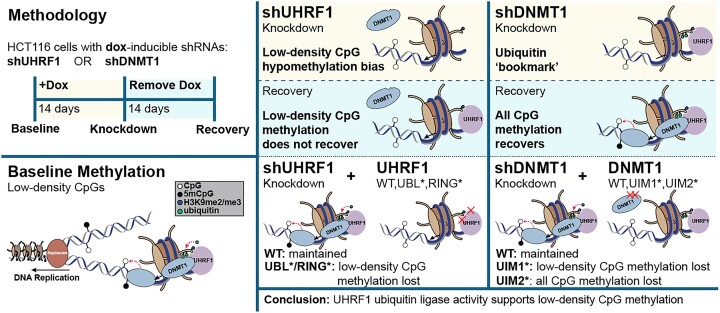

## Introduction

Copying DNA methylation patterns onto newly synthesized DNA is a vital process in all dividing mammalian cells. Cytosines within CpG dinucleotides are the primary substrate for DNA methylation and are disproportionally distributed across the genome. Regions of high CpG density (i.e. CpG islands) account for ∼20% of all CpG sites and primarily occur in and around gene promoters and regulatory elements, while regions of low CpG density frequently occur in intergenic and intronic regions (1,2). In normal cells, CpG islands are largely unmethylated, while the remaining ∼80% of CpGs throughout the genome are methylated (3,4). An epigenetic hallmark of nearly all human cancers is a reversal of this normal DNA methylation pattern, where CpG island promoters acquire DNA hypermethylation associated with gene silencing of tumor suppressor genes (TSGs), while the remainder of the genome becomes hypomethylated (5–7).

Despite a longstanding appreciation for intergenic DNA hypomethylation in cancer, the cause, function and disease relevance of this epigenetic hallmark are not known. These DNA methylation losses induce formation of partially methylated domains (PMDs) that occur almost exclusively in repressive and transcriptionally silent ‘B’ and ‘I’ compartments (8,9). These higher-order chromatin compartments are characterized by repressive histone post-translational modifications (PTMs) like lysine 9 tri-methylation and lysine 27 tri-methylation on histone H3 (H3K9me3 and H3K27me3) (8–10), nuclear lamina association (7), late replication timing (11) and low CpG density (12). PMDs emerge in differentiated cells, are present across tissue types, and deepen in cancer cells and aging fibroblasts that undergo mitotic divisions (9–11,13–16). Understanding how PMDs form is key to understanding the consequences of PMDs in cancer.

DNA methylation patterns are established early in development by the *de novo* methyltransferases DNMT3A and DNMT3B and are primarily maintained in pluripotent and somatic cells by DNMT1. DNMTs rely on histone and non-histone protein interactions to facilitate their epigenetic regulatory functions. This is exemplified by the role of UHRF1 [ubiquitin like with plant homeodomain (PHD) and RING finger domains 1] in support of DNMT1-dependent maintenance methylation (17–19). The RING E3 ubiquitin ligase UHRF1 is a multivalent epigenetic reader and writer that engages with chromatin via histone and DNA interactions (20). The linked tandem Tudor (TTD) and PHD finger of UHRF1 binds histone H3 through interactions with H3K9me2/me3 and the first four amino acids in the H3 N-terminal tail, respectively (21–24). These histone binding domains also interact with DNA ligase 1 (LIG1) and SNF2 DNA helicase LSH to indirectly recruit UHRF1 to chromatin (25–27). Engagement of H3K9me2/me3 enhances UHRF1 binding to hemi-methylated DNA (a DNA replication intermediate) through its SET- and RING-associated (SRA) domain (28,29), and these interactions are necessary to direct its ubiquitin ligase activity towards H3K18, and to a lesser extent H3K14 and H3K23, on H3 tails (30,31). These sites of mono-ubiquitination are binding sites for DNMT1 through tandem ubiquitin interacting motifs (UIMs) embedded in its replication foci targeting domain (RFTS) (32–35). Once recruited to chromatin, DNMT1 processively transfers methyl groups from S-adenosyl-methionine (SAM) to hemi-methylated CpG dinucleotide substrates, copying parent strand DNA methylation patterns to the newly replicated daughter strand (36–39). While the link between UHRF1-mediated ubiquitination and DNMT1 chromatin targeting is established, the extent to which DNMT1 requires ubiquitin signaling through UHRF1 for maintenance DNA methylation remains unclear.

To address this knowledge gap, we comparatively profiled the genome-wide contributions of UHRF1, DNMT1 and their writer and reader domain functions, to the maintenance of CpG methylation using parallel genetic complementation approaches that cover inducible gene knockdowns with wild-type (WT) or domain loss-of-function mutant covers. We show that low-density CpGs are most prone to DNA hypomethylation in the absence of UHRF1. This loss of low-density CpG methylation contributes to the accelerated formation and deepening of PMDs and a reshaping of the DNA methylome that can be reversed with reintroduction of DNMT1, but not UHRF1. We further show that DNMT1-dependent DNA methylation maintenance, independent of CpG density, requires its ubiquitin reading function, and that maintenance methylation at low-density CpG sites relies specifically on UHRF1 E3 ubiquitin ligase activity. Finally, we show that DNA re-methylation following acute DNMT1 depletion relies on UHRF1 and its ubiquitin ligase activity. Collectively, these studies demonstrate that DNA methylation maintenance is a ubiquitin-regulated process involving (but not exclusive to) UHRF1 enzymatic activity and suggest that a disrupted UHRF1-DNMT1 ubiquitin signaling axis contributes to the development of PMDs in human cancers.

## Material and methods

### Reagents

Restriction enzymes used for cloning included: XhoI (Catalog #: R0146S), AgeI (Catalog #: R3552S) and EcoRI (Catalog #: R3101S) from New England Biolabs (NEB, Ipswich, MA, USA). Commercial kits used for the study included: Monarch DNA Gel Extraction Kit (Catalog #: T1020S, NEB, Ipswich, MA, USA), QIAprep Spin Miniprep kit (Catalog #: 27 104, Qiagen, Germantown, MD, USA), High Sensitivity Qubit Fluorometric Quantification (Catalog #: Q32854, Invitrogen, Carlsbad, CA, USA), EZ DNA Methylation Kit (Catalog #: D5002) and EZ DNA Methylation-Gold Kit (Catalog #: D5005) from Zymo (Irvine, CA, USA), CEGX TrueMethyl Whole Genome Kit [Catalog #: CEGXTMWG, v3.1, CEGX (now Biomodal), UK], KAPA Hyper Prep Kit (Catalog # KR0961) and Kapa Illumina Library Quantification qPCR (quantitative polymerase chain reaction) assays (Catalog #: KR0405) from KAPA Biosystems (Wilmington, MA, USA), Agilent DNA High Sensitivity chip (Catalog #: 5067–4626, Agilent Technologies, Inc., Santa Clara, CA, USA), QuantiFluor dsDNA System (Catalog #: E2671, Promega Corp., Madison, WI, USA) and Alpha Screen Histidine detection kit (Catalog #: 6760619M, Revvity, Waltham, MA, USA). Antibodies used for western blotting included: β-actin (Catalog #: 4970 Cell Signalling Technology, Danvers, MA, USA), Beta-tubulin (Catalog #: 66240–1-Ig, Proteintech, Rosemont, IL, USA), DNMT1 (Catalog #: ab134148, Abcam, MA, USA), UHRF1 (Catalog #: 12 387, Cell Signalling Technology, Danvers, MA, USA) and Rabbit IgG horseradish peroxidase-conjugated secondary antibody (Catalog #:GENA934, Sigma-Aldrich, MA, USA). Antibodies used for Chromatin Immunoprecipitation included: H3K9me3 (Catalog #: 39 161, Active Motif, Carlsbad, CA, USA) and H3K27me3 (Catalog #: C36B11, Cell Signaling Technologies, Danvers, MA, USA). Non-standard chemicals and reagents used in the study included: doxycycline (dox) (hyclate) (Catalog #: 14 422, Cayman Chemical, Ann Arbor, MI, USA), 16% methanol-free formaldehyde solution (Catalog #: 28 906, Thermo Fisher Scientific, Waltham, MA, USA), X-tremeGENE HP DNA Transfection Reagent (Catalog #: XTGHP-RO, Roche, Indianapolis, IN, USA), Protein Assay Dye Reagent Concentrate (Catalog #: 5 000 006) and Precision Melt Supermix for High Resolution Melt (HRM) Analysis (Catalog #: 1 725 112 from Bio-Rad, Hercules, CA, USA), Dynabeads Protein G magnetic beads (Catalog #:10004D, Invitrogen, Carlsbad, CA, USA), Herring Sperm DNA (Catalog #: D7290, Sigma-Aldrich, MA, USA), KAPA Pure Beads (Catalog #:KK8000, KAPA Biosystems, Wilmington, MA, USA) and GSK-3483862 (Catalog #: CT-GSKMI-714; Chemietek, Indianapolis, IN, USA). Specialized commercial chips and instruments used in the study included: Illumina NovaSeq6000 sequencer, X Ten sequencer, iScan system and Infinium MethylationEPIC BeadChIP v1.0 and v2.0 (Illumina, San Diego, CA, USA), and a Covaris E220 evolution (Covaris, Woburn, MA, USA).

### Biological resources

Cell lines used in the study included HCT116 (CCL-247), HEK 293T (CRL-3216) and Phoenix AMPHO (CRL-3213) (ATCC, Manassas, VA, USA). Stable competent *Escherichia coli* (High Efficiency) (Catalog # C3040I) and BL21 (DE3) Competent *E. coli* (Catalog #: C2527I) were used for cloning (NEB, Ipswich, MA, USA). Plasmids used in the study included: pMXs-IRES-blasticidin retroviral vector (Catalog #: 72 876), psPAX2 (Catalog #: 12 260), pMD2.G (Catalog #: 12 259), Tet-pLKO-puro (Catalog #: 21 915, Addgene, Watertown, MA, USA) and pQE-80L (Catalog #: 32 943, Qiagen, Germantown, MD, USA). H3K14ub (Catalog #: 16–0398) and H3K18ub (Catalog #: 16–0401) nucleosomes were obtained from Epicypher (Durham, NC, USA).

### Generation of HCT116 cells with doxycycline (dox)-inducible shRNAs (short hairpin ribonucleic acid)

*Tet-pLKO-puro vector cloning:* Empty Tet-pLKO-puro plasmid (40) was purchased from Addgene (#21 915), and shRNA oligos for insertion (Table 1) were synthesized by Eurofins. Oligos were reconstituted in water at a concentration of 100 μM, diluted in 10× annealing buffer [1 M NaCl, 100 mM Tris (pH = 7.4)], brought to 100°C for 10 min and naturally cooled to 30°C to anneal the oligo pair. Annealed oligos were diluted 1:400 in 0.5× annealing buffer. Tet-puro-pLKO plasmid was digested with AgeI and EcoRI (NEB) restriction enzymes, gel-purified (NEB), and ligated to the annealed shRNA oligos. Ligated plasmids were transformed into stable competent *E. coli* (High Efficiency) cells (NEB) and incubated overnight at 37°C on ampicillin Luria Broth (LB) agar plates. Clones were selected and grown in liquid LB culture with ampicillin, and the plasmid was purified by miniprep (Qiagen). Purified plasmids were screened for the shRNA oligo insert using XhoI digestion and sequenced for validation.

**Table 1.** Oligo sequences for shRNA cloning

Oligo	Sequence (5′→ 3′)	Target	TRC ID
shUHRF1_Top	CCGGGCCTTTGATTCGTTCCTTCTTCTCGAGAAGAAGGAACGAATCAAAGGCTTTTT	3′UTR	TRCN0000273256
shUHRF1_Bottom	AATTAAAAAGCCTTTGATTCGTTCCTTCTTCTCGAGAAGAAGGAACGAATCAAAGGC	3′UTR	TRCN0000273256
shDNMT1_Top	CCGGGAGGTTCGCTTATCAACTAATCTCGAGATTAGTTGATAAGCGAACCTCTTTTT	3′UTR	TRCN0000232751
shDNMT1_Bottom	AATTAAAAAGAGGTTCGCTTATCAACTAATCTCGAGATTAGTTGATAAGCGAACCTC	3′UTR	TRCN0000232751

*Lentiviral production for dox-inducible shRNA integration:* Lentivirus production for the stable integration of dox-inducible shRNAs was conducted in HEK 293T cells purchased from ATCC. HEK 293T cells were grown in Dulbecco’s modified Eagle’s medium supplemented with 10% Fetal Bovine Serum (FBS) to ∼70% confluency on 60 mm cell culture plates prior to transfection at 37°C with 5% CO_2_. Cells were then transfected with the Tet-pLKO-puro plasmids targeting either *UHRF1* or *DNMT1* transcripts and the accompanying lentiviral packaging (psPAX2) and envelope (pMD2.G) plasmids with Opti-MEM and Xtreme HP Gene transfection reagent (Roche) per the manufacturer’s protocol. After 15 h of incubation, media was refreshed for an additional 24 h of incubation. Media containing lentiviral particles was then removed, collected and stored at 4°C. Media was refreshed for a second 24-h incubation, and then collected and pooled with the first viral media collection. Viral media was cleared of cell debris by centrifugation at 700 × *g* for 5 min followed by passage through a 0.45 micron filter (Avantor PES 25 mm 0.45 μm). Virus was aliquoted and stored at −80°C prior to transduction.

*Transduction and clonal selection of HCT116 cells:* HCT116 cells were purchased from ATCC and maintained in McCoy’s 5A Media with 10% FBS and 1% penicillin/streptomycin at 37°C with 5% CO_2_. HCT116 cells were plated in six-well plates and infected with media containing 8 μg/ml polybrene (with no antibiotics) and 500 μl of shRNA lentivirus. Media was refreshed 24 h post-infection, and the cells were allowed to grow for an additional 24 h prior to puromycin selection (2 μg/ml) for 2 days. An uninfected plate of HCT116 cells was grown in parallel to test puromycin resistance in the infected cells. Clonal populations for both shUHRF1 and shDNMT1 HCT116 cell lines were isolated using serial dilution of 5000 cells in 96-well plates. Wells containing single cells were noted and allowed to propagate until a sufficient number of cells for freezing and maintenance were attained.

### Generation of HCT116 dox-inducible shRNA cell line with UHRF1/DNMT1 transgene covers

We selected HCT116 dox-inducible shUHRF1 and shDNMT1 clonal cell populations (Clone 9 and Clone 3, respectively) that demonstrated the deepest loss in DNA methylation with dox-inducible knockdown of the endogenous target protein. Selection of these clones allowed us to deplete endogenous protein with dox and query DNA methylation maintained in the presence of WT or mutant transgene protein covers.

*Retrovirus production for UHRF1/DNMT1 WT and mutant transgene covers:* UHRF1, DNMT1 and respective mutants (Table 2) were cloned into the pMXs-IRES-blasticidin retroviral vector (a gift from David Sabatini, Addgene #72 876) by EcoRI and XhoI restriction sites without an affinity tag. Phoenix AMPHO cells (purchased from ATCC) were transfected with pMXs-IRES-blasticidin retroviral vectors with Opti-MEM and Xtreme XP Gene transfection reagent per the manufacturer’s protocol and incubated at 37°C for 24 h. Phoenix AMPHO cells already contain the viral genes necessary for making a virus, therefore only the plasmid with the gene of interest needs to be transfected. Media containing retroviral particles was collected and stored at 4°C. Media was refreshed for a second 24-h incubation, and then collected and pooled with the first viral media collection. Viral media was cleared of cell debris by centrifugation at 700 × *g* for 5 min followed by passage through a 0.45 micron filter (Avantor PES 25 mm 0.45 μm). Virus was aliquoted and stored at −80°C prior to transduction.

**Table 2.** Plasmid constructs for UHRF1/DNMT1 transgene expression

Plasmid–cover–mutant	Mutated domain
pMXs-empty	N/A
pMXs-UHRF1-WT	N/A
pMXs-UHRF1-F46V	UBL
pMXs-UHRF1-F59V	UBL
pMXs-UHRF1-Y188A	Tandem-Tudor
pMXs-UHRF1-G448D	SRA
pMXs-UHRF1-H741A	RING
pMXs-DNMT1-WT	N/A
pMXs-DNMT1-P378A/L381A	UIM1
pMXs-DNMT1-I442A/I487A	UIM2
pMXs-DNMT1-P378A/L381A-I442A/I487A	UIM1 & UIM2 (dblUIM)
pMXs-DNMT1-C1226S	Catalytic

*Transduction of HCT116 dox-inducible shUHRF1 and shDNMT1 cells:* The respective shUHRF1 and shDNMT1 HCT116 cells were plated in six-well plates and infected with media containing 8 μg/ml polybrene (with no antibiotics) and 1 ml of transgene cover retrovirus. Media was refreshed 24 h post-infection, and the cells were allowed to grow for an additional 24 h prior to blasticidin selection (8 μg/ml). An uninfected plate of HCT116 cells was grown in parallel to test blasticidin resistance for the infected cells, and the infected cells were maintained under blasticidin to maintain transgene cover expression.

### HCT116 dox-inducible shRNA time-course

*Dox treatment:* Dox (Cayman) was dissolved in dimethyl sulfoxide (DMSO) to a final concentration of 5 mg/ml, aliquoted and stored at −20°C in complete darkness (dox is light sensitive). For each 2-week time-course, an aliquot of dox was removed from the −20°C, thawed at room temperature and used for the duration of the experiment. In between treatments, dox (5 mg/ml) was stored at 4°C in complete darkness. To treat cells, dox (5 mg/ml) was diluted in phosphate-buffered saline (PBS) (Gibco) to a final concentration of 10 ng/μl and then added to the cell culture media at a final concentration of 10 ng/ml. For the NoDox control, an equivalent amount of DMSO was applied. Dox treatments were refreshed every 2 days throughout the duration of the knockdown time-course.

*Time-course:* For all dox-inducible time-course experiments, the respective HCT116 shRNA cell line was passaged into two separate 10 cm cell culture plates for parallel growth in the absence (Baseline) and presence (Knockdown) of dox (10 ng/ml). Following 2 weeks of treatment, dox was washed out and no longer added to the media. The parallel plates were maintained for an additional 2 (Recovery) to 6 weeks (for HCT116 shUHRF1 Cl.3 and Cl.6) with collection of genomic DNA and protein lysates throughout. HCT116 shGFP cell line for determining dox associated effects was a gift from Stuart Aaronson (41).

### HCT116 recovery time-course with DNMT1 inhibition and UHRF1 transgene cover expression

*Time-course*: HCT116 dox-inducible shUHRF1 Clone 9 cells expressing empty vector, UHRF1 WT, or various loss-of-function UHRF1 mutants were treated with dox (20 ng/ml) for 48 h to reduce expression of endogenous UHRF1 (via shRNA) while maintaining UHRF1 transgene expression. All cell lines were then treated with a single-dose of the non-nucleoside DNMT1 inhibitor, GSK-3484862 (GSK-862) at 500 nM. After 72 h, GSK-862 was washed out in order to recover DNMT1 expression and measure DNA methylation recovery in the presence of various UHRF1 transgene covers. Dox treatment (20 ng/ml every 2–3 days with media changes) was continued for the remainder of the time-course (28 days) to maintain depletion of endogenous UHRF1. Genomic DNA was harvested at Days 0 (prior to GSK-862 treatment), 3, 13 and 28 and submitted for EPIC array(v2) (42) [EPIC array(v1) was retired prior to this experiment] processing at the VAI Genomics Core.

### Western blotting

Cells lysates were prepared as previously described (43). Briefly, cells were lysed in cytoskeleton (CSK) buffer [10 mM PIPES (pH = 7.0), 300 mM sucrose, 100 mM NaCl, 3 mM MgCl_2_, 0.1% Triton X-100, universal nuclease and protease inhibitor cocktail [Roche cOmplete Mini tablets, ethylenediaminetetraacetic acid (EDTA)-free] on ice and centrifuged to remove insoluble components. Lysates were quantified and normalized using the Bradford assay (Bio-Rad), and total protein was size-separated by sodium dodecyl sulphate-polyacrylamide gel electrophoresis (SDS-PAGE) before semi-dry western blot transfer to polyvinylidene fluoride (PVDF) membrane. Membranes were incubated with primary antibodies against β-Actin (1:1 000; Cell Signaling Technology 4970; RRID:AB_2223172), Beta-tubulin (1:50 000; Proteintech 66240–1-Ig), DNMT1 (1:1000; Abcam ab134148) and UHRF1 (1:1 000; Cell Signaling Technology 12 387; RRID:AB_2715501) for 1 h. Following three washes in PBS-T, membranes were incubated with HRP-conjugated secondary antibody (1:10 000; Sigma-Aldrich GENA934, RRID:AB_2722659) for 1 h at room temperature. Membranes were washed again in Phosphate Buffered Saline with Tween 20 (PBS-T), incubated with enhanced chemiluminescence (ECL) substrate and imaged with film.

### DNA isolation

Cells were lysed overnight at 37°C in 2 ml of Tris-EDTA (TE) SDS buffer [10 mM Tris-HCl (pH = 8.0), 0.1 mM EDTA, 0.5% SDS], supplemented with 100 μl of 20 mg/ml proteinase K. DNA was purified by phenol:chloroform extraction in three phases: (1) 100% phenol, (2) phenol:chloroform:isoamyl alcohol (25:24:1), and (3) chloroform:isoamyl alcohol (24:1). For each phase, the aqueous layer was combined with the organic layer in a 1:1 ratio. Samples were quickly shaken, allowed to sit on ice for approximately 5 min, and then separated by centrifugation at 1693 × *g* for 5 min at 4°C. The top aqueous layer was then transferred to a new tube for the next organic phase. Following extraction, DNA was precipitated with 1/10 volume 3 M sodium acetate pH 4.8 and 2.5 volumes 100% ethanol and stored overnight at −20°C. Precipitated DNA was pelleted by centrifugation at 17 090 × *g* for 30 min at 4°C. The pelleted DNA was washed twice with 70% ethanol, allowed to dry for 15 min and resuspended in TE buffer [10 mM Tris-HCl (pH = 8.0), 0.1 mM EDTA]. Samples were then treated with 1 mg/ml RNAse A at 37°C for 30 min and then re-purified by ethanol precipitation as described.

### HRM analysis for DNA methylation

The EZ DNA Methylation Kit (Zymo D5002) was used to bisulfite convert 500 ng of DNA per sample according to the manufacturer’s protocol. Bisulfite converted DNA was eluted in 10 μl of M-elution buffer from the kit and brought up to 54 μl total with DNase-Free water. 5 μl of the bisulfite converted DNA was combined with 10 μl of Precision Melt Supermix for HRM Analysis (BioRad 1 725 112) and 2 μl of Forward and Reverse primers (2 μM stock) (Table 3) and brought to 20 μl with DNase-Free water. A BioRAD CFX Opus93 Real-Time PCR System was used to amplify the DNA at a 60°C annealing temp for 39 cycles and then perform a melt analysis from 65 to 95°C with 0.1°C /10 sec increments. The melt temp (Tm) at the maximum reported RFU value was reported for each amplicon. An amplicon from an unmethylated gene, *RPL30*, was used to control for bisulfite conversion. Genomic DNA isolated from HCT116 DKO1 cells (44) was used as a control for determining relative degree of hypomethylation.

**Table 3.** HRM primer sequences

Primer	Sequence (5′ → 3′)	Region
RPL30_HRM_F	TAATTTAGAAGAGATAGAGAATAGGATAGGAATTTTAG	Promoter
RPL30_HRM_R	ACCATCTTAACGACTACTATTAATAAATAAACTCCTAC	‘’
SFRP1_HRM_F	AGGGGTATTTAGTTGTTGGTTTGTTG	Promoter
SFRP1_HRM_R	CTTCTACACCAAACCACCTCAATA	‘’
Chr6PCH_HRM_F	GGGTTATTTCGTAGGAGGGAGGTTGTTATAGTTTTG	Pericentric Heterochromatin
Chr6PCH_HRM_R	CCTCAATACGCCATTCTCTACTCCCCAAAACC	‘’
STC2_HRM_F	AAGAGAATTGTAGGATGTATGTTTGG	Gene Body
STC2_HRM_R	ACCTAACACCCTACCTAACAACATC	

### Infinium MethylationEPIC BeadChip (EPIC array)

Genomic DNA was quantified by High Sensitivity Qubit Fluorometric Quantification (Invitrogen), and 1.5 μg of genomic DNA was submitted to the Van Andel Institute Genomics Core for quality control analysis, bisulfite conversion and DNA methylation quantification using the Infinium MethylationEPIC BeadChIP v1 or v2 (Figure 5 only) (Illumina) processed on an Illumina iScan system following the manufacturer’s standard protocol (45,46).

### EPIC array data processing

All analyses were conducted in the R statistical software (v4.0.4) (**R Core Team**). Raw IDAT files for each sample were processed using the Bioconductor package ‘SeSAMe’ (v1.8.12) for extraction of probe signal intensity values, normalization of probe signal intensity values, and calculation of β-values from the normalized probe signal intensity values (47–49). The β-value is the measure of DNA methylation for each individual CpG probe, where a minimum value of 0 indicates a fully unmethylated CpG and a maximum value of 1 indicates a fully methylated CpG in the population. CpG probes with a detection *P*-value > 0.05 in any one sample were excluded from analyses.

### Whole genome bisulfite sequencing sequencing and processing

200 ng of DNA was bisfulfite converted using the EZ DNA Methylation-Gold Kit (Zymo, #D5005) according to manufacturer’s instructions. Input DNA was spiked with unmethylated lambda DNA (0.5%) (Promega, #D1521). Replicate bisulfite libraries were generated with the CEGX TrueMethyl^®^ Whole Genome Kit (CEGX, #CEGXTMWG, v3.1) according to manufacturer’s instructions. Libraries were sequenced on the Illumina X Ten. Sequencing reads from whole genome bisulfite sequencing (WGBS) data were aligned to the human genome using v1.2 of an internally developed pipeline Meth10X (50). This is publicly available and can be downloaded from https://github.com/luuloi/Meth10X. Total methylation levels (mC) were calculated by dividing the sum of all C calls with the sum of all C + T calls, and CpGs with a minimum coverage of 5 were used for downstream analyses. Methylation domains (PMD, LMR, UMR) were called using MethylSeekR (51) (v1.0). Highly methylated domain (HMD) domains were called using the ‘complement’ function in bedtools (v2.25.0) against all the coordinates of PMDs, LMRs, and UMRs and were included if the average DNA methylation (average mC) was $ \ge$ 0.8 across the domain.

### Determining differentially hypomethylated CpGs

Differential methylation was calculated within parallel plated experiments (for both bulk and clonal populations) as either Δβ-value or ΔmC for EPIC array and WGBS, respectively, where

*EPIC array*


\begin{equation*}\Delta \beta{\mathrm{valu}}{{\mathrm{e}}_{{\mathrm{Knockdown}}}} = \beta{\mathrm{valu}}{{\mathrm{e}}_{{\mathrm{Knockdown}}}}-\beta{\mathrm{valu}}{{\mathrm{e}}_{{\mathrm{Baseline}}}}\end{equation*}

\begin{equation*}{\mathrm{\Delta\beta}}{\mathrm{valu}}{{\mathrm{e}}_{{\mathrm{Recovery}}}} = \beta{\mathrm{valu}}{{\mathrm{e}}_{{\mathrm{Recovery}}}}-\beta{\mathrm{valu}}{{\mathrm{e}}_{{\mathrm{Baseline}}}}\end{equation*}

*WGBS*


\begin{equation*}\Delta {\mathrm{mCvalu}}{{\mathrm{e}}_{{\mathrm{Knockdown}}}} = {\mathrm{m}}{{\mathrm{C}}_{{\mathrm{Knockdown}}}}-{\mathrm{m}}{{\mathrm{C}}_{{\mathrm{Baseline}}}}\end{equation*}

\begin{equation*}\Delta {\mathrm{mCvalu}}{{\mathrm{e}}_{{\mathrm{Recovery}}}} = {\mathrm{m}}{{\mathrm{C}}_{{\mathrm{Recovery}}}}-{\mathrm{m}}{{\mathrm{C}}_{{\mathrm{Baseline}}}}\end{equation*}

Unless noted, we considered Δβ-value/ΔmC $ \le$ −0.3 as differentially hypomethylated as this is interpreted as roughly 30% reduction in DNA methylation relative to the baseline CpG methylation value.

### Enrichment bias calculation and hypergeometic distribution testing

CpGs (both EPIC and WGBS data) were mapped to their genomic coordinate (hg38) and were then annotated to their genomic annotation relationship (e.g. promoter-TSS, exon) using HOMER (52) (v4.10.3). All enrichment bias calculations were normalized to the distribution of all highly methylated CpGs (within individual bulk and clonal cell populations) on the EPIC array (β-value_Baseline_$ \ge$ 0.85) and WGBS (mC_Baseline_$ \ge$ 0.85), respectively.

Enrichment bias calculations were done by first determining the following values for each feature:

*q* = Number of CpGs that are differentially methylated in feature (e.g. low-density CpGs)

*m* = Total number of highly methylated CpGs that match feature (e.g. all highly methylated low-density CpGs)

*n*= Total number CpGs that do not match feature (e.g. all highly methylated high-density CpGs)

*k* = Total number of all differentially mCpGs (e.g. low-density CpGs + high-density CpGs)

Next, the expected number of CpGs that would be differentially methylated in that feature by random chance was determined with the following equation:


\begin{equation*}e = \;\left( {\frac{m}{{m + n}}} \right)k\end{equation*}

Finally, percent enrichment bias was calculated with the following equation:


\begin{equation*}\% \;{\rm enrichment}\;{\rm bias} = \;\left( {\frac{{q - e}}{k}} \right) \times 100\end{equation*}

where positive or negative enrichment values indicate more or less enrichment for a feature than would be expected by random chance, respectively.

Hypergeometric distribution testing for determining significance of enrichment bias was performed using the phyper() function in R with the following values: *q,m,n,k*.

### Repli-seq integration

Sixteen-phase RepliSeq data (measuring replication timing from early to late replication) for HCT116 was downloaded (53) (GEO: GSE137764) and each phase of replication timing was separated into their own genomic coordinates (both bed and bigwig files) for use in integrative DNA methylation analysis.

### Methylation array analysis of publicly available data

Raw idat files were downloaded from the Gene Expression Omnibus under accession GSE68379 (additional cell lines) and GSE118970 (HCT116/RKO UHRF1 mutants). All analyses were conducted in the R statistical software (v4.0.4) as described under **EPIC array data processing**.

### Chromatin immunoprecipitation

*Cell Fixation and Collection:* Approximately 10 million HCT116 shUHRF1 Clone 3 and Clone 6 NoDox cells were grown to 80% confluency in a 10 cm plates. Cells were washed in the plate with 5 ml of 1× PBS at room temperature. The 1× PBS wash was removed, and 5 ml of Fixing Buffer [(50 mM HEPES-KOH (pH = 7.6), 100 mM NaCl, 1 mM EDTA (pH = 8.0), 0.5 mM ethylene glycol-bis(β-aminoethyl ether)-N,N,N′,N′-tetraacetic acid (EGTA) (pH = 8.0)] was added. Cells were fixed by adding 313 μl of freshly prepared 16% methanol-free formaldehyde solution (Thermo Scientific Catalog #28 906) to a final concentration of ∼1%, and cells were incubated on a shaker at room temperature for 10 min. Formaldehyde was quenched by adding 266 μl of 2.5 M Glycine (final concentration of 125 mM), and cells were incubated for an additional 5 min on the shaker at room temperature. Cells were collected from the plate by scraping the monolayer and diluting the fixation solution with 10 ml ice-cold 1× PBS. Cells were pelleted at 200 × *g* for 5 min at 4°C. Cells were washed twice more with 5 ml ice-cold 1× PBS and collected by centrifugation at 200 × *g* for 5 min at 4°C. The final PBS wash was carefully aspirated from the cell pellet, and cells were flash frozen in liquid nitrogen and stored at −80°C until use.

*Nuclei isolation and sonication:* Cells were thawed on ice for 10 min prior to cell lysis. Cells were lysed in 1 ml LB1 [50 mM HEPES-KOH (pH = 7.6), 140 mM NaCl, 1 mM EDTA, 10% glycerol, 0.5% NP-40, 0.25% Triton X-100, protease inhibitor cocktail (Roche cOmplete Mini tablets, EDTA-free)] and incubated for 10 min rotating at 4°C. Intact nuclei were collected by centrifugation at 1700 × *g* for 5 min at 4°C. Supernatant was removed so as not to disturb the nuclei pellet, and nuclei were resuspended and washed in 1 ml LB2 [10 mM Tris-HCl (pH = 8.0), 1 mM EDTA, 0.5 mM EGTA, 200 mM NaCl, protease cocktail inhibitor] for 10 min rotating at 4°C. Nuclei were collected by centrifugation at 1700 × *g* for 5 min at 4°C, and the supernatant was removed so as not to disturb the nuclei pellet. Finally, nuclei pellets were gently rinsed twice without disturbing the pellet with 1 ml LB3 [10 mM Tris-HCl (pH = 8.0), 1 mM EDTA, 0.5 mM EGTA, 0.01% NP-40, protease cocktail inhibitor] and collected at 1700 × *g* for 5 min at 4°C. Following the two rinse steps, nuclei were resuspended in 1 ml LB3 and transferred to a 1 ml milliTUBE (Covaris) for shearing. Nuclei were lysed and chromatin was sheared to range of 300–600 base-pair fragments using a Covaris E220 evolution Focused ultrasonicator with the following parameters: Peak power (140.0), Duty Factor (5.0), Cycles/Burst (200), Duration (600 s), Temperature (4°C).

*Immunoprecipitation (IP):* Sheared chromatin was quantified by Bradford Assay, and 300 μg of chromatin was brought to a final volume of 500 μl in LB3, and then an additional 500 μl of ChIP Cocktail Mix [40 mM Tris-HCl (pH = 7.6), 150 mM NaCl, 1 mM EDTA (pH = 8.0), 1% Triton X-100, 0.5% NP-40, protease inhibitor cocktail] was added to bring the final volume to 1 ml. Prepared chromatin was then pre-cleared by incubation with 20 μl of pre-washed Dynabeads Protein G magnetic beads (Invitrogen Catalog #: 10004D) for 3 h at 4°C with constant rotation. Prior to incubation with antibody, 10 μl of pre-cleared chromatin was removed and set aside to serve as 1% input. Pre-cleared chromatin was removed by magnetic separation, transferred to a new tube, and immunoprecipitated with either 5 μl of H3K9me3 antibody (Active Motif 39 161, Lot# 14 418 003) or 5 μl of H3K27me3 antibody [Cell Signaling C36B11, Lot#:97 335 (14)] overnight at 4°C with constant rotation. Protein G magnetic beads (35 μl/IP) were blocked overnight in 1 ml of 1× PBS, 0.5% bovine serum albumin (BSA) and 20 μg of Herring Sperm DNA (Sigma Catalog #D7290) at 4°C with constant rotation. The next morning, blocked beads were washed three times with 1× PBS + 0.5% BSA, and two times with WB1 [50 mM Tris-HCl (pH = 7.6), 150 mM NaCl, 5 mM EDTA (pH = 8.0), 0.5% NP-40, 1% Triton X-100]. Antibody-chromatin complexes were incubated with blocked beads for 3 h at 4°C with constant rotation. Unbound chromatin was then removed using magnetic separation, and the beads were washed as follows: three times with WB1, three times with WB2 [50 mM Tris-HCl (pH = 7.6), 500 mM NaCl, 5 mM EDTA (pH = 8.0), 0.5% NP-40, 1% Triton X-100], two times with WB1 and one time with Low Salt TE [10 mM Tris-HCl (pH = 8.0), 1 mM EDTA (pH = 8.0), 50 mM NaCl]. Each wash was a 5-min incubation at 4°C with constant rotation followed by magnetic separation and removal of buffer.

*Elution and DNA clean-up:* To elute DNA from the magnetic beads, 50 μl of elution buffer [10 mM Tris-HCl (pH = 8.0), 10 mM EDTA, 150 mM NaCl, 5 mM Dithiothreitol (DTT), 1% SDS] was added to the beads and incubated at 65°C for 15 min. The elution step was repeated, and eluates combined. Eluents and 1% input (with 90 μl of elution buffer added) were incubated overnight at 65°C with constant shaking to reverse crosslink protein:DNA complexes. The next morning, 2 μl of DNase-free RNase A (10 mg/ml stock) was added to eluents and incubated at 37°C for 1 h. Next, 10 μl of Proteinase K (20 mg/ml stock) was added to eluents and incubated at 37°C for 2 h. DNA was isolated following standard KAPA Pure Beads (KAPA Biosystems Catalog # KK8000) protocol with a 1.5× ratio of beads to DNA. Final elution was in 20 μl of nuclease-free water, and DNA concentration was measure by Qubit dsDNA High Sensitivity Assay kit (Thermo Fisher Scientific Catalog #: Q32851).

*Library preparation:* Immunoprecipitated fragments and saved inputs were quantified by High Sensitivity Qubit Fluorometric Quantification (Invitrogen), and 10 ng of purified DNA for each IP and input sample were used for library preparation with the KAPA Hyper Prep Kit (Part#: KR0961) for these samples. Library preparation including fragment end-repair, A-tail extension, and adapter ligation was conducted per the manufacturer’s instructions (KAPA). Adapter-ligated fragments were amplified with 11 cycles following the recommended thermocycler program, and DNA was purified with two rounds of purification using KAPA Pure Beads (#KK8000). Quality and quantity of the finished libraries were assessed using a combination of Agilent DNA High Sensitivity chip (Agilent Technologies, Inc.), QuantiFluor^®^ dsDNA System (Promega Corp., Madison, WI, USA) and Kapa Illumina Library Quantification qPCR assays (Kapa Biosystems). Individually indexed libraries were pooled and 50 bp, paired end sequencing was performed on an Illumina NovaSeq6000 sequencer using an S2, 100 bp sequencing kit to a minimum read depth of 50 M read pairs per IP library and 100 M read pairs per Input library. Base calling was done by Illumina RTA3 and output of NCS was demultiplexed and converted to FastQ format with Illumina Bcl2fastq (v1.9.0).

### ChIP-seq processing and analysis

ChIP sequencing reads were 3′ trimmed and filtered for quality and adapter content using TrimGalore (v0.5.0) and quality was assessed by FastQC (v0.11.8). Reads were aligned to human genome assembly hg38 with bowtie2 (v2.3.5) and were deduplicated using removeDups from samblaster (54) (v.0.1.24). Aligned BAM files were used for quality control analysis with deeptools (55) (v3.2.0) ‘plotFingerprint’ and ‘plotPCA’ functions. As both H3K9me3 and H3K27me3 domains are broad, we called peaks using Enriched Domain Detector with default parameters (56). For HCT116 shUHRF1 Clone 3 and Clone 6 cells (NoDox, baseline), H3K9me3 and H3K27me3 peak coordinates that were consistent between biological replicates were used for downstream analysis. For shUHRF1 Clone 9 cells and shDNMT1 bulk cell populations, peak coordinates that were consistent between both Clone 3 and Clone 6 (NoDox, baseline) cells were used. For HCT116 H3K36me3 distributions, we used publicly available data (ENCSR091QXP, GSE95914).

### Integrative genomic analysis

To integrate WGBS DNA methylation data with genomic annotations of interest [Replication timing (Repli-seq), H3K9me3/H3K27me3/H3K36me3 distributions (ChIP-seq)], bigwig files for mC-values and ΔmC-values (WGBS) were generated for each sample and time-point for all CpGs, high-density CpGS, and low-density CpGs. Bed files with genomic coordinates for H3K9me3, H3K27me3 and Repli-seq phases were generated as described above. Integrated analyses were conducted with deeptools (55) (v3.2.0) by constructing matrices with ‘computeMatrix’ across queried genomic coordinates with the respective bigwig data and calculating the average mC-values and ΔmC-values with ‘plotProfile’ and ‘plotHeatmap’.

### Methyl domain distribution overlap analysis

Genomic coordinates for Methyl Domains across samples and time-points were determined as described in the section ‘Whole genome bisulfite sequencing sequencing and processing’. Genomic coverage of HMDs, PMDs, LMRs and UMRs were calculated by summation of the length (in base pairs) for each respective domain within each individual sample. To determine transition coverage, Baseline HMD and PMD genomic coordinates were intersected with bedtools (57) (v2.25.0) ‘intersect’ command with the genomic coordinates for Methyl Domains in the Knockdown and Recovery samples, and the length of the intersected domains were summed. Additional overlap analyses among the Methyl Domain genomic locations were conducted using the ‘jaccard’ command from bedtools.

### Calculation of neighbor and methylated CpG density

Neighbor CpG density and neighbor methylated CpG (mCpG) density were calculated as previously described (58). Briefly, neighbor CpG density was calculated by determining the number of CpGs flanking a hypomethylated CpGs (central CpG) $ \pm$ 100 bp, and then dividing by 200 (length of the region). Neighbor mCpG density was calculated by summing the mC-values of the neighboring CpGs. The central CpG was excluded from all calculations.

### NOMe-seq data integration

NOMe-seq data for parental HCT116 cells was downloaded from GEO (GSM1416976). Genomic coordinates were lifted from hg19 over to hg38 using CrossMap (v0.7.0) (59). Bed files with genomic coordinates for highly methylated CpGs (mC-values_Baseline_$ \ge$ 0.85) were generated for both low- and high-density CpGs across the shUHRF1 WGBS datasets. Integrated analyses were conducted with deeptools (55) (v3.2.0) by constructing matrices with ‘computeMatrix’ across queried genomic coordinates for highly methylated CpGs with the respective bigwig data and calculating the average nucleosome occupancy values (100 – GpC (Guanine-Cytosine dinucleotide) methylation) with ‘plotProfile’.

### Protein purification

Recombinant UHRF1 and DNMT1 were generated and purified as full-length proteins with N-terminal 6x-His-MBP (maltose binding protein) tags as previously described (60). DNMT1 RFTS domain WT and mutants were cloned into a pQE-80L plasmid with an N-terminal 6x-His-MBP tag. Plasmids were transformed into BL21 DE3 *E. coli* and grown at 37°C to an OD_600_ of 1.0, followed by addition of 0.2 mM IPTG and induction overnight at 16°C. Induced cultures were harvested and bacteria were resuspended in lysis buffer [50 mM N-[2-Hydroxyethyl]piperazine-N′-[2-ethanesulfonic acid] (HEPES) (pH = 7.5), 250 mM NaCl, 20 mM imidazole, 30 uM ZnOAc, 1 mM PMSF, 1 mM DTT]. Cells were lysed by addition of lysozyme and sonication. Lysate was cleared by centrifugation at 38 000 × *g* at 4°C for 30 min. Lysates were incubated with His60 Ni Superflow resin (Takara Bio) at 4°C with rotation for 1 h. Resin was washed 3× with at least 10 volumes of lysis buffer followed by elution of bound proteins by addition of five volumes of elution buffer [25 mM HEPES (pH = 7.5), 100 mM NaCl, 250 mM imidazole, 1 mM DTT]. Protein purity was assessed by SDS-PAGE and Coomassie staining. Purified proteins were dialyzed into storage buffer [25 mM HEPES (pH = 7.5), 100 mM NaCl, 10% glycerol, 1 mM DTT], concentrated via centrifugal filtration, snap frozen in small aliquots and stored at −80°C.

### Fluorescence polarization

Fluorescence polarization binding assays were performed as described (61). Briefly, recombinant UHRF1 was titrated into assay buffer [25 mM HEPES (pH = 7.5), 100 mM NaCl, 0.05% NP-40] containing 10 nM fluorescent DNA oligonucleotides [5′-FAM-CCAT**X**(5mC)G**X**TGAC-3′ where **X** was interchanged with specific flanking nucleotides (**G**CG**C**, **C**GC**C**, **C**CG**G**; **A**CG**T**, **A**CG**A**, **T**CG**A**)]. Measurements were performed in 384-well plates with triplicate 25 μl reactions and plotted as change in anisotropy.

### Construction of H3K23ub mononucleosomes

H3 ‘K23ub’ thioether mimic was synthesized following a method similar to that described by Hann *et. al* (62). Briefly, a maleimide moiety was covalently conjugated to the C-terminus of ubiquitin (Ub-mal). This Ub-mal was then conjugated to H3 K23C through a stable thioether linkage. The H3 ‘K23ub’ thioether mimic was then assembled into histone octamers and subsequently mononucleosomes. The syntheses of intermediates and final products are described below.

*Synthesis of ubiquitin-thioester (UbTE):* Ubiquitin-GyraseA-His_6_ (UbGyrA) was expressed in Rosetta *E. coli* (RDE3) and induced at 16°C overnight. The pellet was resuspended in lysis buffer [50 mM potassium phosphate monobasic, 300 mM NaCl, 20 mM imidazole, 1 mM PMSF (pH = 8.0)] and lysed using a rod sonicator. The lysate was cleared by centrifugation and the supernatant was loaded onto Ni-NTA beads. The beads were then washed three time with wash buffer [50 mM potassium phosphate monobasic, 1 M NaCl, 20 mM imidazole, 1% NP-40, pH = 8.0)] two times with lysis buffer, and eluted in 1× PBS, 500 mM imidazole, 1 mM TCEP (pH = 7). Solid MESNa was added to the eluate to a final concentration of 500 mM and the pH was readjusted to 7. The reaction was nutated at 4°C for 48 h and splicing was monitored by LCMS. The reaction was then dialyzed against 1% AcOH, the precipitate was removed by centrifugation, and the supernatant was lyophilized. The crude ubiquitin-thioester (UbTE) was purified by reversed-phase high pressure liquid chromatography (HPLC) on a 0–70% buffer B gradient (HPLC solvent A = 0.1% Trifluoroacetic acid (TFA) in ddH_2_O, HPLC solvent B = 90% Acetonitrile (ACN), 10% ddH_2_O, 0.1% TFA). Pooled fractions were lyophilized and stored at −80°C.

*Synthesis of ubiquitin-acyl hydrazide (Ub-NHNH_2_):* UbTE was dissolved in 1× PBS, 6 M guanidine (pH = 5), and hydrazine was added to a final concentration of 500 mM. The reaction was incubated at 30°C for 30 min and then immediately diluted five-fold into HPLC solvent A. The Ub-NHNH_2_ was then purified by reversed-phase HPLC on a 0–70% solvent B gradient. Pooled fractions were lyophilized and stored at −80°C.

*Synthesis of ubiquitin-N-(2-aminoethyl)maleimide (Ub-mal):* Ub-NHNH_2_ was dissolved in oxidation buffer [0.2 M phosphate, 20 mM NaNO_3_, 6 M guanidine (pH = 3)] and nutated at 4°C to form the acyl-azide *in situ*. An equal volume of 200 mM N-(2-aminoethyl)maleimide dissolved in 1.5 M phosphate pH 7.4 was added to the acyl-azide and the reaction was nutated at room temperature for 15 min to form the Ub-mal. The reaction was diluted five-fold into HPLC buffer A and Ub-mal was purified by reversed-phase HPLC on a 0–70% solvent B gradient. Pooled fractions were lyophilized and stored at −80°C.

*Synthesis of H3 K23ub thioether mimic:* An excess of H3 K23C was combined with Ub-mal and dissolved in 1× PBS (pH = 6.8). The reaction was nutated at 4°C overnight and conversion was monitored by LCMS. Upon complete consumption of free Ub-mal, the reaction was diluted five-fold into HPLC solvent A and H3K23ub purified by reversed-phase HPLC on a 30–70% solvent B gradient. Pooled fractions were lyophilized and stored at −80°C.

*Histone octamer assembly:* Histones were dissolved in unfolding buffer [20 mM tris, 6 M guanidine, 0.5 mM EDTA, 10 mM TCEP (pH = 7.5)] and combined in a 1:1:0.95:0.95 molar ratio (H2A:H2B:H3K23ub:H4). The pooled histone solution was diluted with unfolding buffer to a final combined histone concentration of 1 mg/ml. The histone mixture was then dialyzed against refolding buffer [10 mM tris, 2 M NaCl, 0.5 mM EDTA, 1 mM TCEP (pH = 7.5)] multiple times to ensure complete buffer exchange. The refolded histone octamers were concentrated and then isolated by purification on a GE Superdex 75 15/300 increase size-exclusion column. Pooled fractions were concentrated, diluted to 50% glycerol and stored at −20°C.

*Mononucleosome assembly:* Mononucleosome assembly was performed according to the previously described salt dilution method with slight modification. Briefly, purified octamers were mixed with Widom-601 DNA (1:1 ratio) in a 2 M salt solution [10 mM Tris (pH = 7.5), 2 M NaCl, 1 mM EDTA, 1 mM DTT]. After incubation at 37°C for 15 min, the mixture was gradually diluted (∼15 min) at 30°C with dilution buffer [10 mM Tris (pH = 7.5), 10 mM NaCl, 1 mM EDTA, 1 mM DTT). The assembled mononucleosomes were concentrated and characterized by native gel electrophoresis (5% acrylamide gel, 0.5× TBE, 120 V, 40 min) using ethidium bromide staining.

### *In vitro* methyltransferase assays

*In vitro* methyltransferase assays were performed by reacting WT and mutant forms of recombinant His-MBP-DNMT1 with ^3^H-labeled S-adenosylmethionine and the previously described biotinylated hemi-methylated Dup_1 DNA substrate (https://doi.org/10.1093/nar/gkt753) (5′-GATmCGCmCGATGmCGmCGAATmCGmCGATmCGATGmCGAT-3′). Briefly, 10 μl reactions were assembled in DNMT1 reaction buffer [20 mM HEPES (pH = 7.5), 50 mM KCl, 1 mM EDTA, 25 μg/ml BSA] containing 150 nM DNMT1, 300 nM DNA and 20 μM ^3^H-SAM. Reactions were incubated at 37°C, then quenched at various timepoints by addition of 190 μl of 20 μM S-adenosylhomocysteine. Reactions were transferred to 96-well Streptavidin FlashPlates (Perkin Elmer) and incubated at room temp for 15 min to bind biotinylated DNA, followed by three washes with 200 μl wash buffer per well [50 mM Tris (pH = 7.4), 0.05% Tween-20]. Scintillation counts were measured using a MicroBeta2 microplate reader (Perkin Elmer). Data was plotted using GraphPad Prism.

### *In vitro* ubiquitin ligase assays

*In vitro* ubiquitin ligase assays were performed as previously described (60). Substrate nucleosomes with 26 bp of linker DNA were purchased from Epicypher.

### Alpha screen assays

Alpha screen assays were performed in 384-well plates using the Alpha Screen Histidine (Nickel Chelate) detection kit from Revvity. H3K14ub and H3K18ub nucleosomes (with native ubiquitin linkages), and unmodified nucleosomes with linker DNA, were purchased from Epicypher. H3K23ub nucleosomes (with dichloroacetone (DCA) mimic ubiquitin linkage) were synthesized as described. WT and mutant recombinant His-MBP-RFTS or His-MBP-UHRF1 and nucleosomes (with 5′ biotinylated DNA) were diluted in alpha assay buffer [25 mM HEPES (pH = 7.5), 250 mM NaCl, 0.05% NP40], mixed in a final volume of 10 μl, and incubated at room temperature for 30 min. Alpha donor and acceptor beads were diluted in alpha assay buffer and added to the reactions. The final reaction volume was 20 μl and the final concentration of each bead was 20 μg/ml. Reactions were incubated at room temperature for 30 min protected from light. Plates were read on an alpha screen compatible Perkin Elmer plate reader. Data was plotted in GraphPad Prism and fitted with nonlinear regression using the specific binding with Hill slope mod.

### Materials availability

All unique/stable reagents (plasmids, cell lines) generated in this study are available from the Lead Contact with a completed Materials Transfer Agreement.

## Results

### Low-density CpGs are most prone to DNA hypomethylation when UHRF1 levels are reduced.

**Figure 1.**
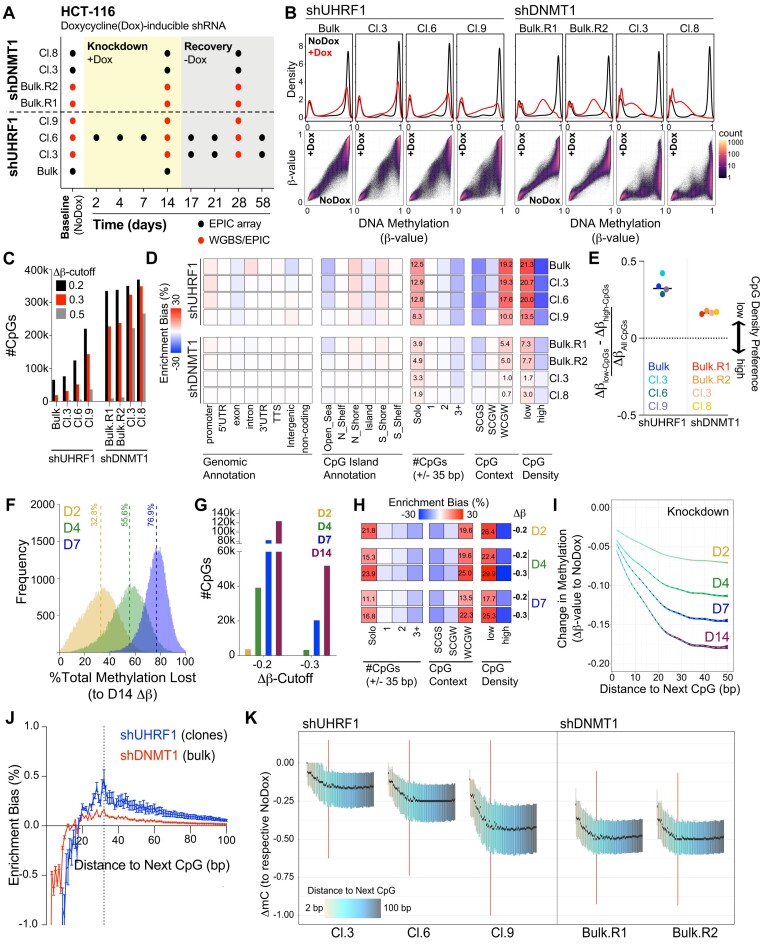
Low-density CpGs are most prone to DNA hypomethylation when UHRF1 levels are reduced. (**A**) Schematic of experimental design including samples, time-points, and types of DNA methylation data collected. (**B**) DNA methylation distributions of EPIC array probes from dox-inducible shRNA HCT116 cell lines without (black) and with (red) dox treatment (10 ng/ml) for 14 days. **Top panel:** Density plots for CpG probe distribution across DNA methylation levels [β-value: 0 (unmethylated) to 1 (methylated)]. **Bottom panel:** Density scatterplots demonstrating density of probes and DNA methylation level in baseline (x-axis) and knockdown (y-axis) methylomes. (**C**) Bar graph for number of differentially mCpGs (EPIC array) for each knockdown experiment (β-value_Baseline_$ \ge$ 0.85; varying Δβ-value_Knockdown_ cutoffs relative to each respective Baseline sample). (**D**) Hypergeometric analysis of significantly hypomethylated CpGs for each sample from **1C**. Positive values indicate significant overrepresentation for hypomethylation of the feature and negative values indicate significant underrepresentation. Enrichment bias values are provided for the most significant positive enrichments. #CpGs indicates the number of CpGs $ \pm$ 35 bp upstream and downstream of the hypomethylated CpG. CpG Context represents the −1/+1 position nucleotide (S = C or G; W = A or T) flanking the hypomethylated CpG. CpG density is determined by the number of bps to the next CpG (either upstream or downstream). Low density $ \ge$ 20 bp, high density < 20 bp. (**E**) Normalized preference for CpG density among the shUHRF1 and shDNMT1 samples. ‘CpG density preference’ is calculated by subtracting the average Δβ-value of all the high-density CpGs (EPIC) from the average Δβ-value of the low-density CpGs, divided by the total Δβ-value of all CpGs. As this is a sample-dependent calculation, the difference in the numerator (Δβ_low_density CpGs_ – Δβ_high_denisty CpGs_) informs on which CpG density is most hypomethylated relative to all hypomethylated CpGs, where negative values indicate high-density CpG preference and positive values indicate low-density CpG preference. (**F**) Histogram of the calculated percent methylation lost [(β-value_Knockdown_**_(X)_** – β-value_Baseline_)/Δβ-value_Knockdown(Day14)_]*100 across early time points (X = Days 2, 4, 7) for all significantly hypomethylated CpGs in shUHRF1 knockdown (Cl.6) from **1C**. Median % Methylation Lost is indicated by the dotted line for each time point. (**G**) Bar graph for number of differentially mCpGs (β-value_Baseline_$ \ge$ 0.85, varying Δβ-value_Knockdown_ cutoffs relative to Baseline) across the early time-points in shUHRF1 knockdown (Cl.6). (**H**) Hypergeometric analysis of significantly hypomethylated CpGs (from **1G**) across the early shUHRF1 (Cl.6) time-points. Legend from **1D** applies. (**I**) Average loss of DNA methylation for all highly methylated CpGs (β-value_Baseline_$ \ge$ 0.85) across ‘Distance to the Next CpG’ binning. Dotted line indicates the average Δβ-value as a function of distance, colored boundaries indicate 95% confidence intervals. (**J**) Hypergeometric analysis of significantly hypomethylated CpGs (WGBS) for each sample across CpG binning by ‘Distance to the Next CpG’ (bp). Positive enrichment bias indicates overrepresentation, negative enrichment bias indicates underrepresentation. Dotted line indicates peak positive enrichment bias for all shUHRF1 clones at 32 bp. (**K**) Boxplots for change in methylation (ΔmC_Knockdown_) of all highly methylated CpGs (mC_Baseline_$ \ge$ 0.85) from WGBS of each indicated sample across CpG binning by ‘Distance to the Next CpG’. The 32 bp boxplot (peak enrichment bias from **1J**) is highlighted. Whiskers were removed for figure clarity. **See also**Supplementary Figure S1.

To enable the comparative study of DNA methylation maintenance dynamics supported by UHRF1 and DNMT1, we engineered HCT116 human colorectal carcinoma cells to express dox-inducible shRNAs targeting the *UHRF1* or *DNMT1* 3′UTRs (Figure 1A and Supplementary Figure S1A). HRM analysis showed that UHRF1 depletion caused progressive loss of DNA methylation at promoters (*SFRP1)*, gene bodies (*STC2*), and pericentric heterochromatin (Chr6) over 2 weeks of dox treatment (Supplementary Figure S1B), while dox treatment alone had no effect (Supplementary Figure S1C). Using the Illumina EPIC array platform (63), we profiled the individual contributions of UHRF1 and DNMT1 to DNA methylation maintenance at ∼800 000 individual CpGs from bulk and clonal cell populations at intervals up to 2 weeks following dox-inducible knockdown (Figure 1A and B and Supplementary Figure S1A). Clear differences in DNA hypomethylation dynamics were observed comparing UHRF1 and DNMT1 knockdown. Notably, DNMT1 knockdown caused hypomethylation of almost all highly methylated CpGs (β-value_Baseline_$ \ge$ 0.85) (Supplementary Figure S1D), while UHRF1 knockdown revealed a sub-population of highly methylated CpGs that were resistant to DNA hypomethylation (Figure 1B and C).

We next asked which CpGs were most prone to DNA hypomethylation (Δβ-value_Knockdown_$ \le$ −0.3) in the absence of UHRF1 or DNMT1. To do this, we classified CpGs by locality (e.g. in genes or regulatory regions, CpG island status) and by CpG density. Two relative CpG density measurements were used. The first CpG density measure is where the number of adjacent CpGs (± 35 bp) and flanking nucleotide sequences (S = C/G, W = A/T) are jointly considered (i.e. ‘Solo-WCGW’) (11). The second defines ‘high-density’ CpGs as the distance between two adjacent CpGs being < 20 bp apart (upstream or downstream), and ‘low-density’ CpGs are those where CpGs are $ \ge$ 20 bp apart. We then performed enrichment bias analysis (normalized to the distribution of CpGs with β-value_Baseline_$ \ge$ 0.85), and we found that CpG density (by both density measurements) was the primary attribute that defined DNA hypomethylation in the absence of UHRF1 (Figure 1D). While DNMT1 knockdowns demonstrated a slight enrichment bias towards low-density CpGs (Figure 1D), normalization of low- to high-density CpG hypomethylation showed that UHRF1 knockdowns consistently had a higher preference for low-density CpG hypomethylation (Figure 1E). These findings were verified by WGBS (50) (Supplementary Figure S1E and F).

To further investigate the relationship between UHRF1 and CpG density, we next asked if UHRF1 preferentially bound CpGs in the ‘Solo-WCGW’ context. We note that a prior study drew a correlation between ‘Solo-WCGW’ DNA methylation maintenance and UHRF1 expression (64). Fluorescence polarization binding assays with DNA probes showed that UHRF1 did not have a ‘WCGW’ sequence bias for binding to hemi-mCpGs (Supplementary Figure S1G). We then considered if the enrichment bias for ‘Solo-WCGW’ could be attributed to an inherent bias present in the human genome for low-density CpGs and A/T flanking nucleotide sequences. Indeed, enrichment bias analysis of all CpGs in the genome showed that ‘WCGW’ CpGs are inherently low-density CpGs, as the likelihood of being a ‘WCGW’ CpG increases with decreasing CpG density (Supplementary Figure S1H and I). Finally, we asked if the average methylation level of low-density CpGs was equivalent to ‘Solo-WCGW’ CpGs across 291 cell lines previously profiled on the Illumina 450K array (65). These data showed that the average β-value of low-density CpGs is highly correlated with the ‘Solo-WCGW’ CpG average DNA methylation level but incorporates over 100 000 additional probes into the analysis (Supplementary Figure S1J). Collectively, these data show that maintenance DNA methylation at low-density CpGs depends on UHRF1, and, while we acknowledge that modifications to histone tails that attract or repel UHRF1 influence its local concentration and may indirectly impact where the UHRF1 SRA engages hemi-methylated DNA in the genome, our data support a model in which the association of UHRF1 with low-density CpG methylation (which biasedly occurs in WCGW sequence contexts) is not the result of a sequence bias in the SRA domain of UHRF1.

To extend this analysis, we next profiled DNA methylation in a clonally expanded, dox-inducible, shUHRF1 cell population (Cl.6) at different time-points following UHRF1 knockdown (Figure 1A and F–I). Consistent with HRM analysis of candidate loci (Supplementary Figure S1B), deepening hypomethylation of CpGs occurred over 2 weeks of UHRF1 knockdown (Figure 1F), especially at low-density CpGs (Figure 1G and H). Importantly, the degree of DNA hypomethylation (Δβ-value_Knockdown_) again was dependent on CpG density (Figure 1I). Given this result, we next asked whether there was an enriched peak distance to the next CpG among the hypomethylated CpGs (from Supplementary Figure S1E). We found an inflection of peak distance enrichment for CpG hypomethylation at 32 bp for the shUHRF1 knockdown clones, an enrichment that was attenuated by DNMT1 knockdown (Figure 1J). Profiling of all highly methylated CpGs (mC_Baseline_$ \ge$ 0.85) by WGBS likewise showed the greatest DNA hypomethylation at low-density CpGs, with a plateau of loss at ∼32 bp (highlighted in red; Figure 1K). Notably, mathematical modeling of DNA methylation maintenance kinetics [derived from Repli-BS-seq (66)] predicted that DNMT1 acts processively on CpGs that are up to 36 bp apart (67). Collectively, these data suggest that maintenance DNA methylation mechanisms are not conserved throughout the genome and that perhaps CpG density influences how DNA methylation is maintained.

### Hypomethylation of low-density CpGs reshapes the DNA methylome in the absence of UHRF1 and DNMT1

**Figure 2.**
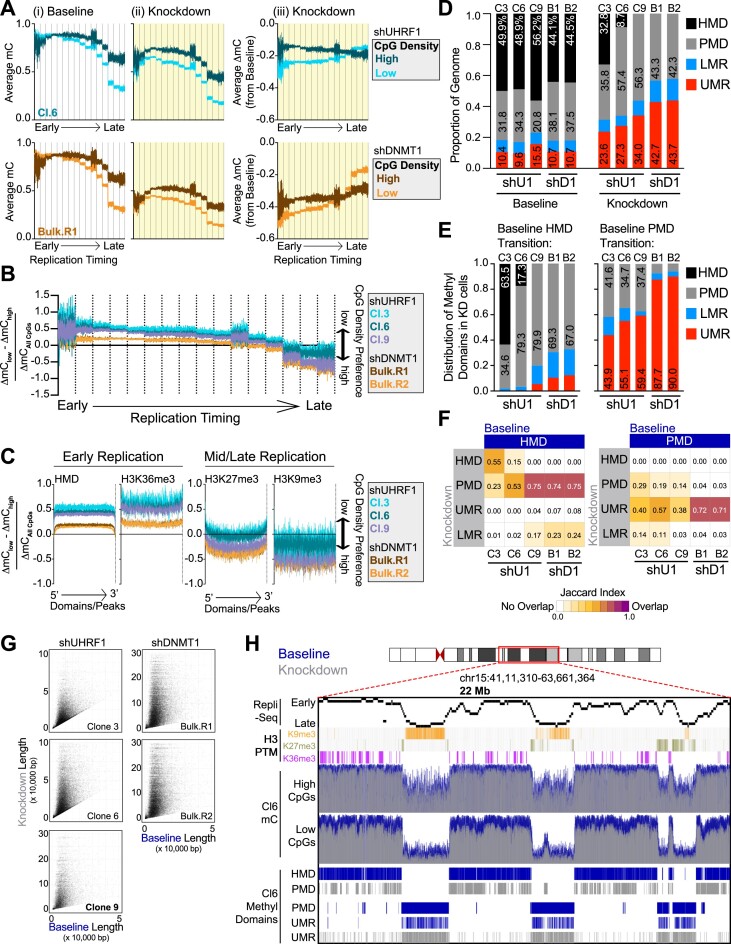
Hypomethylation of low-density CpGs in the absence of UHRF1 and DNMT1 reshapes the DNA methylome. (**A**) Average DNA methylation (mC) of high and low density CpGs in (i) Baseline and (ii) Knockdown phases and the (iii) average change in methylation (ΔmC_Knockdown_) across each replication timing phase (16-phase) in HCT116 cells. Replication timing phases are assigned in 50 kb windows genome-wide, and the average DNA methylation (from WGBS) is calculated in 100 bp bins from the start of the 50 kb window to the end for each timing phase. Vertical lines indicate the separation of the different replication timing phases from early to late. shUHRF1 Cl.6 and shDNMT1 Bulk (replicate 1) samples are provided in the main figure. Remaining WGBS samples are presented in Supplementary Figure S2A. (**B**) Normalized preference for CpG density in shUHRF1 and shDNMT1 samples across replicating timing phases. ‘CpG Density Preference’ is calculated by subtracting the average ΔmC of all the high-density CpGs (WGBS) from the average ΔmC of the low-density CpGs, divided by the total ΔmC of all CpGs in 100 bp bins as described in **2A**. Positive values indicate preference for low-density CpGs and negative values for high-density CpGs. (**C**) Normalized preference for CpG density in shUHRF1 and shDNMT1 samples across genomic features known to be localized in early replicating chromatin (HMDs, H3K36me3) and mid/late replicating domains (H3K27me3/H3K9me3). DNA methylation is averaged from the 5′ end of the Domain/Peak to the 3′end in size normalized windows. (**D**) Proportional coverage of the genome for called Methylation Domains in the Baseline and Knockdown stage for each shUHRF1 and shDNMT1 WGBS sample. HMD, highly methylated domain; PMD, partially methylated domain; LMR, lowly methylated region; UMR, unmethylated region. Baseline mC distributions for methylation domains are provided in Supplementary Figure S2C. (**E**) Distribution shift of called Methylation Domains in the Knockdown methylome from Baseline HMDs (left) and PMDs (right). (**F**) Overlap analysis of called Methylation Domains from Baseline and Knockdown methylomes across shUHRF1 and shDNMT1 WGBS samples. The Jaccard Index measures the extent of the overlap with 0 being no overlap to 1 being complete overlap. (**G**) Scatterplot of UMR length in Baseline methylomes versus Knockdown methylomes. Over 90% of the UMRs located within Baseline PMDs expand with loss of UHRF1 and DNMT1. (**H**) Browser shot of 22 Mb region on Chr15 demonstrating the observations made from WGBS sequencing analysis of Baseline and Knockdown methylomes in the shUHRF1 Cl.6 sample integrated with Repli-Seq, histone PTMs, and locality of called methylation domains. **See also**Supplementary Figure S2.

Hypomethylation at low-density CpG sites is linked to the cellular mitotic index and the formation of PMDs, especially in late replicating chromatin (11,15). Indeed, it has been hypothesized that the increased mitotic index in cancer shortens the available time for cells to complete methylation maintenance before the next cell division, resulting in passive hypomethylation in late replicating genomic regions. Our data are consistent with this hypothesis, in that HCT116 colorectal carcinoma cells are hypomethylated at low-density CpGs in late replicating genomic regions at baseline, while high- and low-density CpGs in early replicating chromatin are hypermethylated [Figure 2A(i) and Supplementary Figure S2A and B(i)].

Given the preferential hypomethylation of low-density CpGs when UHRF1 is absent, we next asked how depleting UHRF1 or DNMT1 affected DNA methylation at high- and low-density CpGs in early- and late-replicating chromatin. As coverage of CpGs is limited with EPIC arrays, we integrated our WGBS data (Figure 1A) from the three shUHRF1 clones (Clone 3, 6, 9) and two shDNMT1 bulk populations (R1, R2) to enable more rigorous analysis of DNA methylation patterns in the context of chromatin features genome-wide. In early replicating chromatin, depleting UHRF1 or DNMT1 induced hypomethylation at high- and low-density CpGs, but the extent of hypomethylation was deeper for low-density CpGs [Figure 2A(iii) and Supplementary Figure S2B(iii)]. UHRF1 knockdowns demonstrated a stronger preference than DNMT1 knockdowns for hypomethylation at low-density CpGs (Figure 2B), similar to what we observed by EPIC array analysis (Figure 1E). In late-replicating chromatin, low-density CpGs had a lower DNA methylation level at baseline than high-density CpGs [Figure 2A(i)], and both UHRF1 and DNMT1 knockdowns drove low-density CpGs to become almost completely demethylated [Figure 2A(ii)]. As low-density CpGs became deeply hypomethylated [Figure 2A(ii)], high-density CpGs in late-replicating chromatin became the preferential target for hypomethylation in the absence of UHRF1 or DNMT1 [Figure 2A(iii) and B]. As an orthogonal analysis, we profiled the CpG density preference for hypomethylation in the absence of UHRF1 or DNMT1 using annotations of histone PTMs and methylation domains known to localize with early (HMDs, H3K36me3) and mid/late replicating chromatin (H3K27me3/H3K9me3) (Figure 2C) (8,9). Indeed, HMDs and H3K36me3-marked chromatin demonstrated increased preference for hypomethylation of low-density CpGs in UHRF1 knockdowns over DNMT1 knockdowns, while H3K27me3/H3K9me3-marked chromatin did not (Figure 2C).

As hypomethylation at low-density CpG sites generates PMDs (11,12,15), we next asked how DNA methylation domains throughout the genome were altered with UHRF1 or DNMT1 knockdown (Supplementary Figure S2C). For both UHRF1 and DNMT1 knockdown, we found substantial restructuring of the DNA methylome where HMDs were lost, and expanded coverage of the genome by PMDs and unmethylated regions (UMRs) emerged (Figure 2D). HMDs predominately transitioned into PMDs (Figure 2E and F) through preferential hypomethylation at low-density CpGs (Figure 2C), while pre-existing UMRs (present within PMDs) expanded in size (Figure 2E–G). Deep hypomethylation adjacent to the original UMR (Supplementary Figure S2D) initiated UMR expansion, with loss of DNA methylation at both high- and low-density CpGs (Supplementary Figure S2E). Collectively, these data show that depleting UHRF1 or DNMT1 substantially reshapes the DNA methylome (Figure 2H), forming PMDs in early-replicating chromatin and expanding UMRs in late-replicating chromatin.

### Recovery of low-density CpG methylation requires UHRF1

Above, we used the dox-inducible knockdown system to understand how DNA methylation patterns change when UHRF1 or DNMT1 are knocked down. Here, we used the same system to understand how the DNA methylome recovers from a hypomethylated state when UHRF1 or DNMT1 are re-expressed (Figure 1A). In this case, we treated the engineered HCT116 human colorectal carcinoma cells with dox for 2 weeks (to generate hypomethylated genomes), terminated the dox treatment and then monitored DNA methylation recovery for 2 weeks as UHRF1 or DNMT1 expression was restored (Supplementary Figure S3A).

**Figure 3.**
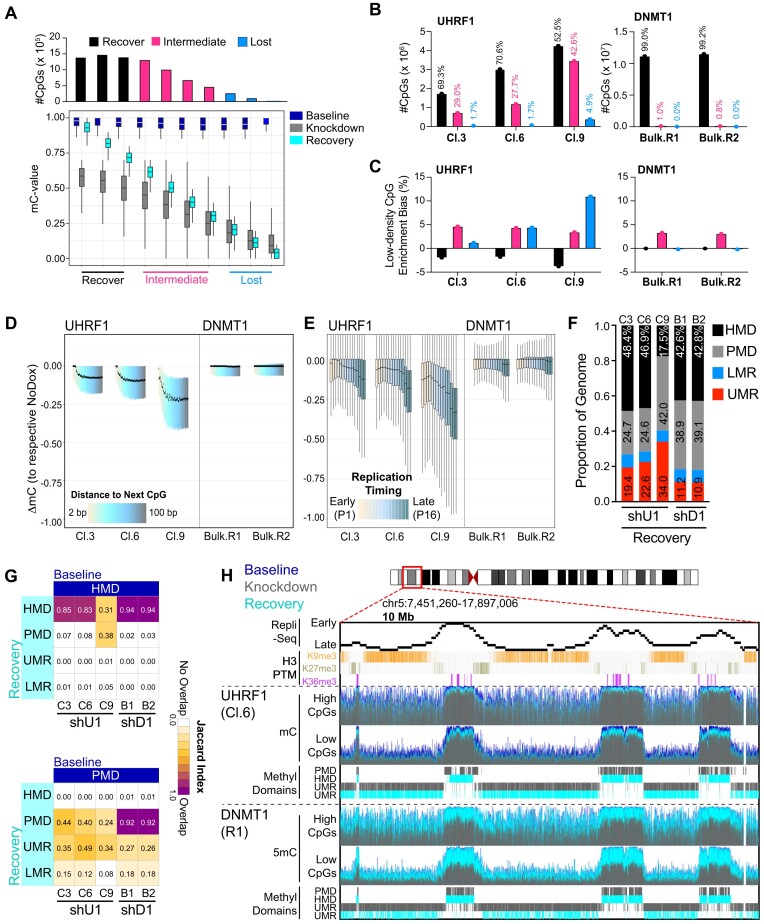
Recovery of low-density CpG methylation requires UHRF1. (**A**) Recovery analysis strategy for classifying recovery dynamics of significantly hypomethylated CpGs (from Supplementary Figure S1D) across shUHRF1 and shDNMT1 (WGBS) samples. shUHRF1 Clone 9 is presented to illustrate recovery dynamic binning analysis. DNA methylation recovery is determined by calculating the ΔmC of Day 28 samples (Recovery) to the respective Baseline (NoDox) samples. DNA methylation recovery measurements were then binned into three groups, where CpGs with recovery values (|ΔmC_Recovery_|*100) < 30% are considered lost, > 70% are considered recovered, and between 30 and 70% are considered intermediate recovered. (**B**) Distribution of recovery categories among shUHRF1 and shDNMT1 samples (WGBS). Legend from **3A** applies. (**C**) Hypergeometric analysis for enrichment of low-density CpGs across recovery categories for each shUHRF1 and shDNMT1 sample (WGBS) from **3B**. (**D**) Boxplots for change in methylation recovery (ΔmC_Recovery_) of all highly methylated CpGs (mC_Baseline_$ \ge$ 0.85) from WGBS of each indicated sample across CpG binning by ‘Distance to the Next CpG’ [color bar (2–100 bp)]. Whiskers were removed for figure clarity. (**E**) Boxplots for change in methylation recovery (ΔmC_Recovery_) of all highly methylated CpGs (mC_Baseline_$ \ge$ 0.85) from WGBS of each indicated sample across replication timing phases. (**F**) Proportional coverage of the genome of called Methylation Domains in the Recovery stage for each shUHRF1 and shDNMT1 sample (WGBS). (**G**) Overlap analysis of called Methylation Domains from Baseline and Recovery methylomes across shUHRF1 and shDNMT1 WGBS samples. The Jaccard Index measures the extent of the overlap with 0 being no overlap and 1 being complete overlap. (**H**) Browser shot of 10 Mb region on Chr5 demonstrating the observations made from WGBS analysis of the Recovery methylome in shUHRF1 Cl.6 and shDNMT1 replicate 1 samples integrated with Repli-Seq, histone PTMs, and locality of called Methylation Domains. **See also**Supplementary Figure S3.

To profile DNA methylation recovery of the most significantly hypomethylated CpGs (from analysis in Supplementary Figure S1E), we compared changes in DNA methylation from the end of the recovery phase (28 days) to the original baseline DNA methylation measurements for each individual CpG (ΔmC_Recovery_). The DNA methylation recovery measurements were then binned into three categories, where CpGs with recovery values (|ΔmC_Recovery_|*100) < 30% were considered to lose DNA methylation (‘Lost’), > 70% were considered to recover DNA methylation (‘Recover’), and between 30 and 70% were considered to intermediately recover DNA methylation (‘Intermediate’) (Figure 3A and Supplementary Figure S3B). Following re-expression of DNMT1, ∼99% of the hypomethylated CpGs were re-methylated to within 70% of their baseline value (Figure 3B). Recovery was almost universal across CpGs of varying densities, although the minority of CpGs that did not recover their baseline methylome (intermediate recovery) were enriched for low-density CpGs (Figure 3C). We obtained similar results using EPIC arrays and clonally expanded, dox-inducible DNMT1 knockdown lines (Supplementary Figure S3C–E). After UHRF1 re-expression, however, CpG re-methylation was variable (Figure 3A and B). Unlike DNMT1 re-expression, UHRF1 re-expression was able to recover ∼50–70% of the hypomethylated CpGs among the shUHRF1 clones, while ∼30–50% of hypomethylated CpGs demonstrated either intermediate recovery or lost DNA methylation completely (Figure 3B and Supplementary Figure S3B). CpGs that did not recover with UHRF1 re-expression (Intermediate/Lost) were significantly enriched for low-density CpGs (Figure 3C). Consistent with these data, profiling of all baseline highly methylated CpGs (β-value_Baseline_$ \ge$ 0.85) demonstrated that DNA methylation recovery was more complete for high-density CpGs while low-density CpGs remained the most demethylated with re-introduction of UHRF1 (Figure 3D). Indeed, low-density CpG methylation never returned to the baseline state, even after an additional 30 days (Day 58) of restored UHRF1 expression (Supplementary Figure S3F and G). Interestingly, the neighboring baseline methylation density flanking hypomethylated CpGs was lower for CpGs with intermediate recovery compared to full recovery (Supplementary Figure S3H), indicating that the methylation density surrounding a CpG influences the ability of the CpG to recover DNA methylation as previously proposed (58).

We next considered how replication timing integrated with DNA methylation recovery dynamics. While DNMT1 re-introduction universally recovered DNA methylation across replication timing phases, UHRF1 re-introduction primarily restored DNA methylation levels in early replicating chromatin but not late replicating chromatin (Figure 3E). UHRF1 re-expression also restored HMDs in early replicating regions of the genome (particularly in Cl.3 and Cl.6), but the expanded UMRs never reverted to their original state (Figure 3F–H). Collectively, these data show that HCT116 cells can fully restore their DNA methylation patterns after DNMT1 loss and re-expression, but that late replicating low-density CpG methylation patterns are not restored after UHRF1 loss and re-expression. These data suggest that UHRF1 creates an epigenetic memory or ‘bookmark’ that DNMT1 uses to re-establish DNA methylation patterns at low-density CpGs.

### UHRF1 ubiquitin ligase activity is required for maintenance of low-density CpG methylation

**Figure 4.**
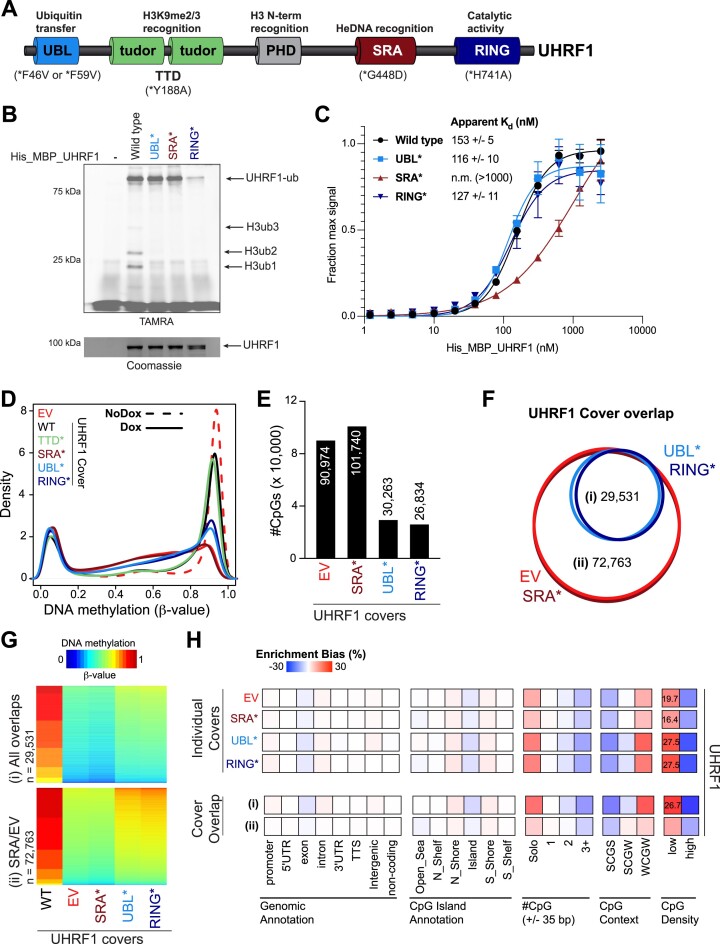
UHRF1 ubiquitin ligase activity is required for the maintenance of low-density CpG methylation. (**A**) Schematic of UHRF1 protein domains and mutations made to the UHRF1 transgene covers. (**B**) *In vitro* ubiquitin ligase activity assays with recombinant WT UHRF1 or the indicated domain loss-of-function mutants and unmodified nucleosomes with 26 bp linker DNA as substrate. (**C**) *In vitro* Alpha-screen proximity assays measuring interactions between unmodified nucleosomes with 26 bp linker DNA and recombinant recombinant WT UHRF1 or the indicated domain loss-of-function mutants. (**D**) Density plot for CpG probe distribution across DNA methylation levels [β-value: 0 (unmethylated) to 1 (methylated)] for WT and mutant(*) UHRF1 transgene covers. (**E**) Bar graph of number of hypomethylated CpGs (EPIC array) for each UHRF1 cover (β-value_WT_cover_$ \ge$ 0.85; Δβ-value_mutant_covers_$ \le$ −0.3). (**F**) Venn diagram demonstrating overlap of hypomethylated CpGs from UHRF1 cover experiments. (**G**) Heatmap of DNA methylation (β-value) values for overlapping hypomethylated CpGs from the (i) Ubiquitin mutant covers and (ii) EV and SRA mutant covers. (**H**) Hypergeometric analysis of hypomethylated CpGs from UHRF1 cover experiments. Positive values indicate significant overrepresentation for hypomethylation of the feature (genomic annotation, CpG island annotation, etc.) and negative values indicate significant underrepresentation. Enrichment bias values are provided for the most significant positive enrichments. **See also**Supplementary Figure S4.

The appreciated substrates for UHRF1 enzymatic activity are mono-ubiquitination sites on histone H3 tails and PAF15, as well as UHRF1 itself (auto-ubiquitination) (68,69). We therefore hypothesized that the ‘bookmark’ required for DNMT1 to mediate recovery of low-density CpG methylation was UHRF1-dependent ubiquitination. To test this hypothesis, WT and various domain loss-of-function mutant UHRF1 transgenes were used. To determine the effect of these mutations on UHRF1 biochemical activities, we tested UHRF1 WT, UBL* (F59V), SRA* (G448D), and RING* (H741A) in enzyme activity and binding assays toward recombinant nucleosomes with extended DNA linkers (Figure 4B-C). All mutations compromised UHRF1 ubiquitin ligase activity toward histone H3 (Figure 4B). Notably, the RING mutant, but not the UBL mutant that perturbs the E2 interaction, abolished UHRF1 auto-ubiquitination (68,69). Despite all three mutants being compromised for H3 ubiquitin ligase activity, only the DNA-binding SRA mutant perturbed UHRF1 interaction with nucleosomes (Figure 4C). The residual binding activity with this mutant can be attributed to its multivalent interaction with the H3 N-terminus (60). However, integration of these data with enzyme activity assay results supports the conclusion that the reduced binding activity of the SRA mutant is sufficient to disrupt UHRF1 targeting to the H3 tail as a substrate for ubiquitination.

Next, UHRF1 WT and loss of function mutants were stably introduced into the dox-inducible UHRF1 shRNA line that showed the deepest loss in UHRF1 expression and DNA demethylation in the knockdown state (Cl.9; Figure 4A and Supplementary Figure S4A). UHRF1 transgene expression in the absence of dox treatment had no effect on DNA methylation (Supplementary Figure S4B). Consistent with prior reports (70–72), a mutation in the aromatic cage of the UHRF1 TTD that disrupts binding to H3K9me2/me3 (Y188A) had no effect on maintenance DNA methylation, while a SRA mutation that abolishes DNA binding (G448D) had the same effect as dox-inducible UHRF1 knockdown cells complemented with an empty vector control (EV) (Figure 4A and D–F). In contrast, UBL (F46V and F59V) (68,69) or RING (H741A) (73) mutations that perturb ubiquitin transfer had partial effects on DNA methylation maintenance (Figure 4A and D–F, and Supplementary Figure S4C). The concordant effects of UBL and RING mutations implicates ubiquitin ligase activity targeted to histone H3, rather than UHRF1 auto-ubiquitination, in the maintenance of this subset of DNA methylation (Figure 4B) (69). We then used enrichment bias analysis to identify the subset of CpGs dependent on UHRF1 ubiquitin ligase activity for DNA methylation maintenance. UHRF1 ubiquitin ligase activity was needed to support DNA methylation maintenance in low CpG density regions of the genome in both HCT116 and RKO colon cancer cell lines (Figure 4E–H and Supplementary Figure S4D–F), consistent with the hypothesis of histone ubiquitination functioning as a bookmark for low-density CpG methylation. Collectively, these data support the conclusion that UHRF1 ubiquitin ligase activity is essential for the maintenance of low-density CpG methylation.

### UHRF1 ubiquitin ligase activity is essential for DNA re-methylation following DNMT1 inhibition

**Figure 5.**
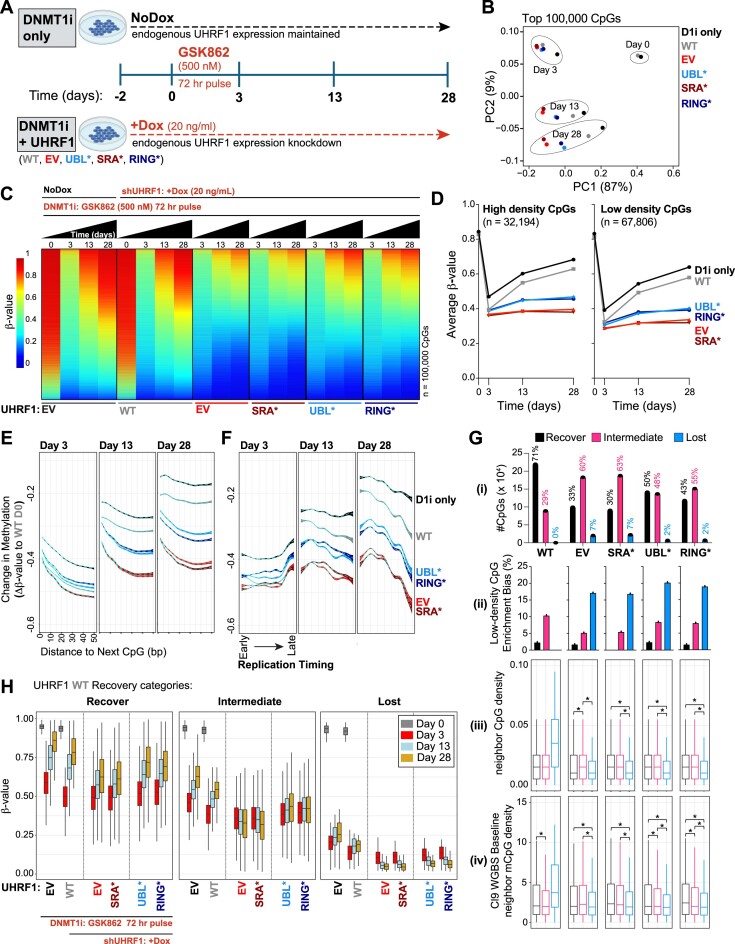
UHRF1 ubiquitin ligase activity is required for recovery of low-density CpG methylation. (**A**) Schematic of treatment paradigm in HCT116 dox-inducible shUHRF1 Clone 9 cells with UHRF1 transgene covers (used in experiments presented in Figure 4). To inhibit DNMT1 activity, all cell lines were treated with a single-dose of GSK-3484862 (GSK862) (500 nM) for 72 h. For the DNMT1i only control (D1i only), cells were not treated with dox in order to maintain expression of endogenous UHRF1. DNMT1i + UHRF1 cover lines were pretreated with dox (20 ng/ml) for 48 h prior to GSK862 to remove expression of endogenous UHRF1 and measure depletion and recovery of DNA methylation in the presence of the UHRF1 transgene cover. Dox treatment (20 ng/ml) was maintained throughout the duration of the experiment (28 days) to maintain knockdown of endogenous UHRF1. Created with BioRender.com (**B**) Principal components analysis of the top 100 000 most variably methylated CpGs (EPIC array) across all samples and time-points. (**C**) Heatmaps of the top 100 000 most differentially methylated CpGs (comparison: Day 28 UBL*/RING* versus Day 28 D1i only/D1i + WT cover). CpGs are ranked from highest to lowest DNA methylation for the Day 28 WT cover cells. (**D**) Average DNA methylation of top 100 000 most differentially methylated high-density CpGs (left) and low-density CpGs (right) from **C** across all time-points. (**E**) Average loss of DNA methylation for all highly methylated CpGs (β-value_(WTcover_Day0)_$ \ge$ 0.85) across bins of decreasing CpG density. Dotted line indicates the average Δβ-value as a function of distance. Boundaries indicate 95% confidence intervals. (**F**) Average loss of DNA methylation for all highly methylated CpGs (β-value_(WTcover_Day0)_$ \ge$ 0.85) across replication timing phases. Dotted line indicates the average Δβ-value as a function of replication timing. Boundaries indicate 95% confidence intervals. (**G**) Characterization of recovery dynamic categories across UHRF1 transgene cover experiments: (**i**) Number of significantly hypomethylated CpGs (β-value_(WTcover_Day0)_$ \ge$ 0.85; Δβ-value_(Day3-WTcover_Day0)_$ \le$ −0.3) that demonstrate indicated recovery dynamics among the UHRF1 cover transgene experiments. **(ii)** Hypergeometric analysis for enrichment of low-density CpGs across recovery dynamic categories for each UHRF1 transgene cover experiment. **(iii)** Boxplots of neighboring CpG density (−/+ 100 bp flanking CpG of interest). *pval < 2.2e-16 by one-sided Mann–Whitney *U*-test. **(iv)** Boxplots of neighboring methylated CpG density (values derived from shUHRF1 Cl.9 baseline WGBS; −/+ 100 bp flanking CpG of interest). *P-value < 2.2e-16 by one-sided Mann–Whitney *U*-test. (**H**) DNA methylation distributions across the time-course for CpGs that recover with UHRF1 WT (left), intermediately recover with UHRF1 WT (middle), or are lost (right). DNA methylation values among the EV and UHRF1 mutant covers for CpGs in the designated UHRF1 WT recovery bins demonstrate which CpGs require UHRF1 for DNA methylation recovery. See also Supplementary Figure S5.

To begin testing this ‘bookmarking’ hypothesis, we performed a time-course experiment using UHRF1 WT and mutant cover HCT116 cell lines studied in Figure 4 to analyze the contribution of UHRF1 ubiquitin ligase activity to the restoration of DNA methylation following acute DNMT1 inhibition. To inhibit DNMT1 activity in this model, we treated cells for 72 h with the DNMT1-specific degrader GSK 3484862 (GSK862) (74,75) and followed the recovery dynamics of DNA methylation in the presence or absence of WT UHRF1 or in the presence of SRA, UBL or RING mutants (Figure 5A). Notably, single treatments of HCT116 cells with 1 μM or 500 nM GSK862 hypomethylated the genome rapidly over 72 h, and, unlike DNMT1 knockdown by shRNA, DNA methylation patterns were not fully restored up to 42 days post-treatment (Figure 3 and Supplementary Figure S5A). The 500 nM dose of GSK862 hypomethylated the genome to a level comparable with DNMT1 knockdown mediated via shRNA (Supplementary Figure S5B), and despite this caveat of incomplete recovery, was chosen moving forward with this experiment. For assessment of DNA methylation recovery dynamics in the presence of UHRF1 WT or mutant transgene covers, we pre-treated cells with dox (20 ng/ml) for 48 h to induce expression of shUHRF1 targeting endogenous UHRF1 expression while maintaining UHRF1 transgene expression. Effects of drug treatments on DNA methylation dynamics at candidate loci as well as DNMT1 and UHRF1 protein expression were validated prior to EPIC array analysis (Supplementary Figure S5C and D).

DNA methylation loss and recovery dynamics in this experimental paradigm were similar in cells supported by endogenous UHRF1 (D1i only) and a WT UHRF1 transgene (D1i + WT) (Supplementary Figure S5E and F). Based on this, D1i only and D1i + WT served as controls for DNA methylation recovery analysis with UHRF1 KD (EV) and mutant covers (SRA*, UBL* and RING*). Principal component and heatmap analyses of the top 100 000 most differentially methylated CpGs showed little variance across samples at Day 3 of the hypomethylation phase (Figure 5B and C). However, samples began diverging during the recovery phase at Day 13 and 28, where the recovery controls (D1i only; D1i + WT) moved closer to baseline DNA methylation measurements at Day 0 compared to EV and mutant covers. Notably, among these 100 000 most differentially methylated CpGs, there was a 2-fold enrichment for low-density CpGs over high-density CpGs, and low-density CpGs demonstrated deeper hypomethylation (Figure 5D). Consistent with our previous analyses (Figure 5B and Supplementary Figure S5E and F), the recovery controls demonstrated highly similar recovery dynamics at both high and low-density CpGs (Figure 5C and D), while DNA methylation recovery in EV and mutant covers was severely compromised.

We next compared DNA methylation recovery dynamics across samples by calculating the change in DNA methylation for all individual UHRF1 covers and time-points to the baseline methylomes of D1i + WT recovery controls (Δβ-value_WT_Day0_). Consistent with our analysis of the most differentially methylated CpGs (Figure 5C and D), EV and UHRF1 mutant covers did not aid DNA methylation recovery to the same extent as the UHRF1 WT cover (Supplementary Figure S5G, top). Notably, the change in DNA methylation at Day 28 was highly correlated between the UBL and RING mutant covers, demonstrating that perturbations to either UHRF1 ubiquitin signaling domain yields similar consequences for DNA methylation recovery (Supplementary Figure S5G, bottom).

Next, we profiled recovery dynamics of all hypomethylated CpGs (Δβ-value_Day3-Day0_ ≤ −0.3, n = 345 426 CpGs) across CpG density as measured by distance to the next CpG in base pairs (Figure 5E). As was observed with shRNA mediated knockdown of UHRF1 or DNMT1 (Figure 1K), all samples demonstrated deepening hypomethylation dependent on CpG density at Day 3 (Figure 5E). In the recovery phase (Day 13 and Day 28), the WT transgene cover was able to recover DNA methylation at low-density CpGs, similar to the D1i only recovery control. However, the ability to recover low-density CpG methylation was not observed with the EV and mutant covers (Figure 5E).

We next asked if the inability of late-replicating chromatin to recover DNA methylation (Figure 3E) was linked to the ubiquitin ligase activity of UHRF1 as was observed with low-density CpG methylation. Consistent with both our UHRF1 and DNMT1 knockdowns (Figure 2A and Supplementary Figure S2A), we observed that acute DNMT1 inhibition (Day 3) hypomethylated CpGs in both early and late-replicating chromatin, with deeper hypomethylation occurring in early (Figure 5F). During the recovery phase (Days 13 and 28), controls consistently recovered more DNA methylation across all replicating timing phases than EV or the UHRF1 mutant covers (Figure 5F). Indeed, CpGs that did not recover DNA methylation in the EV and UHRF1 cover mutants were particularly enriched in late replicating chromatin (Supplementary Figure S5H), consistent with our previous observation with UHRF1 knockdown (Figure 3E). Notably, the UHRF1 WT cover recovered DNA methylation in early-replicating chromatin substantially better than the UBL or RING mutants (Figure 5F).

Next, we binned recovery dynamics at Day 28 into ‘recover, intermediate, and lost’ categories as described previously (Figure 3A). While the UHRF1 WT cover recovered over 70% of hypomethylated CpGs (Δβ−value_Day3-Day0_$ \le$ −0.3), UBL and RING mutant covers only recovered ∼43–50% and EV and SRA mutant covers had the least amount of recovery at ∼30% [Figure 5G(i)]. Consistent with the conclusion that UHRF1 ubiquitin ligase activity is required for low-density CpG methylation recovery, we observed a significant enrichment for low-density CpGs in the intermediate and lost recovery bins for UBL and RING mutant covers [Figure 5G(ii)].

We next considered the density of CpGs and methylated CpGs flanking ($ \pm$ 100 bp) the hypomethylated CpGs [Figure 5G (iii,iv)]. While the neighbor CpG density was not significantly different between fully and intermediately recovered CpGs [Figure 5G(iii)], the baseline methylated CpG density was significantly lower for CpGs with intermediate recovery rather than full recovery among the WT, UBL* and RING* covers [Figure 5G (iv)]. This observation is consistent with previous reports that suggest that neighboring methylated CpG density influences the recovery of DNA methylation at CpGs with imprecise/unfaithful maintenance through ‘neighbor-guided correction’ (58,76). Indeed, it has been suggested that DNA hypomethylation in PMDs occurs through loss of ‘neighbor-guided correction’ (77); however, the role of UHRF1 for neighbor-guided correction remains unknown.

To dissect the contribution of UHRF1 to ‘neighbor-guided correction’, we focused on the recovery dynamic binning of the UHRF1 WT cover (Figure 5H). First, CpGs that recovered DNA methylation with either endogenous UHRF1 or UHRF1 WT cover also demonstrated the ability to recover DNA methylation with either loss of UHRF1 (EV) or with mutant UHRF1 covers (Figure 5H, left), indicating that these CpGs do not require UHRF1 to recover DNA methylation. Conversely, CpGs that demonstrated intermediate recovery with either endogenous UHRF1 or UHRF1 WT cover did not have intermediate recovery with EV or the mutant covers, including perturbation of the UBL or RING domains (Figure 5H, middle). Importantly, the neighboring mCpG density of CpGs that fully recovered with UHRF1 WT cover were significantly higher than CpGs that demonstrating intermediate recovery [Figure 5G(iv)]. Collectively, these results support a model in which CpGs that fully recover do so through neighbor-guided correction while CpGs that have intermediate recovery cannot restore DNA methylation through neighbor-guidance, but rather rely on the ubiquitin ligase activity of UHRF1. Taken together, these results show that UHRF1 ligase activity is required for the maintenance and recovery of low-density CpG methylation following acute DNMT1 depletion. These data support a ‘bookmarking’ function for UHRF1 ubiquitin ligase activity in DNA maintenance and recovery phases with a bias towards support of low-density CpG (re)methylation.

### DNMT1 ubiquitin recognition is essential for DNA methylation maintenance

**Figure 6.**
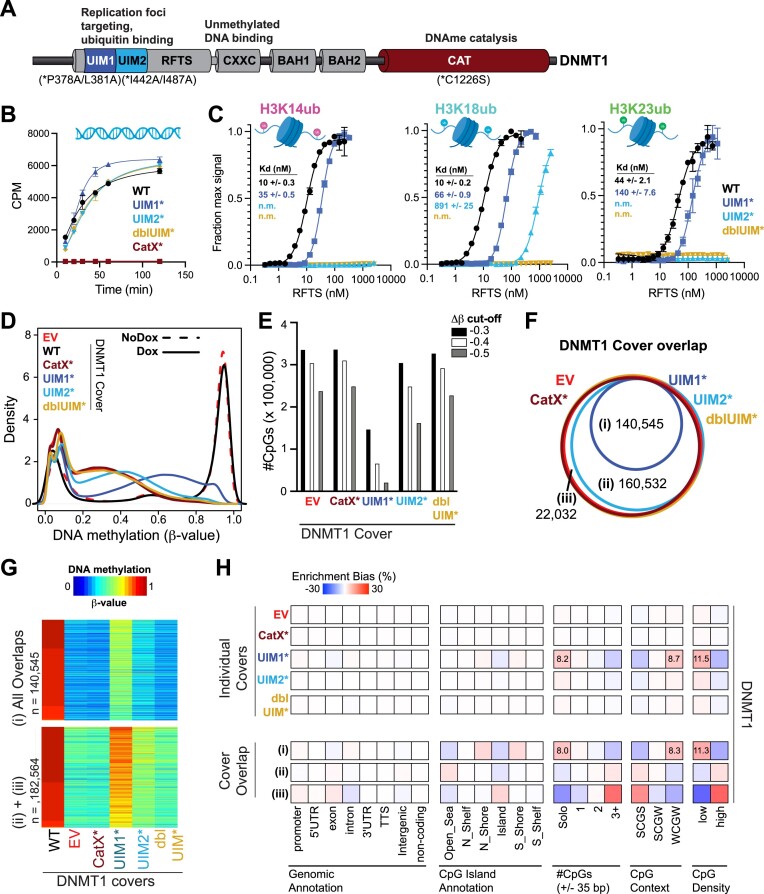
DNMT1 ubiquitin recognition is essential for DNA methylation maintenance. (**A**) Schematic of DNMT1 protein domains and mutations made to the DNMT1 transgene covers. (**B**) *In vitro* methyltransferase assays measuring catalytic activity of recombinant full length DNMT1 WT, Catalytic Dead (CatX), and UIM mutants toward free hemi-methylated DNA. (**C**) *In vitro* Alpha-screen proximity assays measuring interactions between H3 ubiquitinated nucleosomes (K14ub, K18ub, K23ub) and recombinant DNMT1 RFTS domain WT and UIM mutants. (**D**) Density plot for CpG probe distribution across DNA methylation levels [β-value: 0 (unmethylated) to 1 (methylated)] for WT and mutant(*) DNMT1 transgene covers. (**E**) Bar graph of number of hypomethylated CpGs (EPIC array) for each DNMT1 cover (β-value_WT_cover_$ \ge$ 0.85; Δβ-value_mutant_covers_$ \le$ −0.3, −0.4, −0.5). (**F**) Venn diagram demonstrating overlap of hypomethylated CpGs from DNMT1 cover experiments. (**G**) Heatmap of DNA methylation (β-value) values for overlapping hypomethylated CpGs from (i) All overlaps and (ii + iii) UIM2, dblUIM, CatX and EV overlaps. (**H**) Hypergeometric analysis of hypomethylated CpGs from DNMT1 cover experiments. Positive values indicate significant overrepresentation for hypomethylation of the feature and negative values indicate significant underrepresentation. Enrichment bias values are provided for the most significant positive enrichments. **See also**Supplementary Figure S6.

To further test the hypothesis that ubiquitin serves as a ‘bookmark’ for DNMT1-mediated DNA methylation maintenance, we next considered the contributions of the DNMT1 UIMs to its DNA methylation maintenance function (Figure 6A). *In vitro* methyltransferase activity assays with recombinant full-length, WT and domain loss-of-function DNMT1 mutants (Figure 6A) showed that UIM mutations had no effect on the catalytic activity of DNMT1 toward free DNA (Figure 6B). However, *in vitro* binding assays with recombinant DNMT1 RFTS domain showed that RFTS binding to H3K14/18/23ub nucleosomes required UIM2 and partially depended on UIM1 (Figure 6C), suggesting that the two UIMs make separate and unequal contributions to DNMT1 recruitment to ubiquitinated nucleosomes. Mutations to both UIMs (dblUIM) completely abolished DNMT1 RFTS binding to ubiquitinated nucleosomes.

Next, we used a genetic complementation strategy (as in Figure 4) to determine how UIM mutants contribute to DNA methylation maintenance (Figure 6A and Supplementary Figure S6A). DNMT1 transgene expression in the absence of dox treatment had no effect on DNA methylation (Supplementary Figure S6A and B), and a WT DNMT1 transgene cover maintained nearly all DNA methylation in the absence of endogenous protein (Figure 6D). Consistent with perturbed binding to ubiquitinated nucleosomes (Figure 6C), mutations to UIM2 (both individual and in dblUIM) abolished the ability of DNMT1 to maintain DNA methylation much like a catalytically dead enzyme (CatX; Figure 6D and E). As the DNMT1 UIM mutants maintain catalytic activity *in vitro* (Figure 6B), these results collectively indicate that DNMT1 requires recruitment to chromatin through ubiquitin interaction to perform DNA methylation maintenance. UIM1 mutation, however, did not completely abolish maintenance methylation (Figure 6D,E). Rather, DNA methylation maintenance was attenuated (Figure 6D,E) much like the ability of DNMT1 RFTS with UIM mutation to bind ubiquitinated nucleosomes (Figure 6C). Indeed, the DNMT1 UIM1 mutant was still able to maintain DNA methylation at > 50% of all highly methylated CpGs (β-value_WT_$ \ge$ 0.85) while mutants in UIM2 and the catalytic domain could not (Figure 6F and G). Maintenance DNA methylation in the absence of UIM1 was most notably affected in regions of low CpG density (Figure 6H), consistent with a role for UHRF1 ubiquitin ligase activity promoting DNMT1 function via recruitment through UIM1. Additionally, data showing that mutation of UIM2 (or both UIMs) completely abolishes DNMT1 maintenance methylation (while inactivation of UHRF1 ubiquitin ligase activity only partially impairs DNMT1 maintenance methylation) suggests that additional E3 ligases function redundantly with (or independent of) UHRF1 to support DNA methylation maintenance elsewhere in the genome. Taken together, these data demonstrate that ubiquitin reading activity is essential for DNMT1-mediated DNA maintenance methylation and that UHRF1 ubiquitin ligase-dependent recruitment of DNMT1 through UIM1 is necessary to support DNA methylation in regions of the genome with low-density CpGs.

## Discussion

In this study, we showed that UHRF1 ubiquitin ligase activity and DNMT1 ubiquitin reader function are essential for the maintenance of low-density CpG methylation. While mutations to the RING and UBL domains of UHRF1 perturbed maintenance methylation of low-density CpGs, these mutants could maintain methylation patterns at higher-density CpGs. Conversely, mutation of the SRA domain (which abolishes the interaction of UHRF1 with DNA) disrupted maintenance DNA methylation of additional CpGs, suggesting that UHRF1 aids DNMT1 in the maintenance of DNA methylation through both ubiquitin-dependent and independent mechanisms. Furthermore, it is clear from the growing body of co-factors [LIG1 (25) and LSH (26,27)] and additional substrates for UHRF1-directed ubiquitination [PAF15 (78,79) and H3 (31,32)], that maintenance DNA methylation is not a conserved process genome-wide but rather a coordinated effort among different mechanisms to ensure the faithful inheritance of DNA methylation prior to cell division.

In support of this notion, profiling of DNA methylation maintenance kinetics [via coupling BrdU/EdU labelling of newly replicated DNA with next-generation sequencing (64,66,80) and mass spectrometry (81)] has made clear that maintenance methylation does not solely occur in the immediate wake of the replication fork, but rather can be divided into replication-fork coupled (rapid) and replication-fork uncoupled (delayed) phases. Additionally, the kinetics of DNA methylation maintenance are not conserved across CpG dinucleotides, as high-density and low-density CpGs demonstrate rapid and delayed methylation rates, respectively (64,66,67,82). Indeed, mathematical modeling of maintenance DNA methylation kinetics [from Repli-BS data (66)] emphasized the importance of CpG density with regard to maintenance methylation rates (67). Remarkably, this modeling predicted that DNMT1 acts rapidly and processively on CpGs that are, on average, within 36 bp of a neighboring CpG (67). The field has converged on the importance of CpG density for maintenance DNA methylation, particularly within a window of 32–36 bps to the next CpG, in several additional ways. First, the ‘solo-WCGW’ hypomethylation signature observed in PMDs defines ‘solo’ CpGs as those lacking additional CpGs within 35 bp upstream or downstream of its location (11). Second, depletion of UHRF1 enriches for hypomethylated CpGs that are ∼32 bp from neighboring CpGs (Figure 1J). Third, the degree by which CpGs are hypomethylated in response to loss of both UHRF1 and DNMT1 deepens with increasing distance to neighboring CpGs but plateaus at ∼35 bps (Figure 1K). Collectively, these data suggest that DNMT1 acts processively if it encounters the next CpG within ∼35 bp. After ∼35 bp, DNMT1 requires additional recruitment mechanisms to ensure fidelity of low-density CpG maintenance methylation. We hypothesize that UHRF1 ubiquitin ligase activity functions as a compensatory mechanism for the lack of DNMT1 processivity/neighbor-guided correction in regions with low CpG density.

### Connecting PMD formation to the maintenance DNA methylation machinery

The implications of low-density CpG hypomethylation can be observed from methylomes of aged and cancerous tissues where PMDs deepen due to loss of DNA methylation maintenance at these CpGs (9,11,83–85). The ‘solo-WCGW’ signature was first described within PMDs where CpGs in this context are most prone to DNA methylation loss (11), and we and others have linked UHRF1 depletion to hypomethylation of these CpGs (64). Our WGBS analysis of the methylome in the absence of UHRF1 revealed that hypomethylation of low-density CpGs that reside in baseline HMDs results in the formation of PMDs. To our knowledge, this is the first study to report a direct perturbation to a DNA methylation writer or co-factor that induces formation of PMDs. To date, PMD formation through loss of low-density CpG methylation has largely been associated with the mitotic index of the cells as PMDs deepen through progressive cell divisions (15). However, the exact mechanism that contributes to PMD formation remains unknown. We hypothesize that UHRF1 dysfunction (specifically to the ubiquitination axis) contributes to the formation of PMDs in ageing and cancer. Importantly, we observe a divergence in the ability of the methylome to recover with re-introduction of UHRF1 as early replicating regions regain their HMD status while late replicating regions with deepened PMDs do not recover. This observation supports the paradigm that the fidelity of DNA methylation maintenance is dependent on replication timing where early replicating chromatin (with both high- and low-density CpGs) is faithfully copied while late replicating chromatin (enriched in low-density CpGs) has limited time to ensure this process is complete before mitosis.

### Disruption of UHRF1 ubiquitin ligase activity is compatible with tumorigenesis

We previously showed that UHRF1 supports the growth and metastasis of human colorectal cancer (CRC) through repression of TSG expression (70). Notably, UHRF1 functional domains do not contribute equally to CRC maintenance. Histone and DNA interactions mediated by the PHD and SRA domains, respectively, are essential for repression of TSGs and oncogenic growth. However, perturbations to the RING domain that induce hypomethylation of low-density CpGs (Supplementary Figure S4F) are dispensable for oncogenic growth and proliferation (70), suggesting that loss of low-density CpG methylation is compatible with tumorigenesis. Indeed, PMDs (through hypomethylation of low-density CpGs) are universally observed across cancer methylomes (11). Our results suggest that the erosion of DNA methylation during oncogenesis (at low-density CpGs) is connected to dysregulated UHRF1 ubiquitin ligase signaling.

### CpG density is a potential substrate specificity determinant for UHRF1 enzymatic activity

In addition to H3 tails, UHRF1 also mono-ubiquitinates the PCNA associated factor PAF15 to facilitate an interaction with DNMT1 (31,32,78,79). It has been proposed that PAF15 ubiquitination serves as a DNMT1 recruitment mechanism in early replicating chromatin, whereas H3 tail ubiquitin recruits DNMT1 in late replication (79). So how is the ubiquitin ligase activity of UHRF1 directed towards certain substrates for maintenance of low-density CpG methylation? *In vitro* ubiquitination assays measuring UHRF1 activity towards semi-synthetic nucleosome substrates provides a potential biochemical explanation for this association between CpG methylation density and UHRF1-dependent H3 ubiquitination (69). Specifically, increasing hemi-methylation density in linker DNA directs UHRF1 enzymatic activity away from histone H3 – presumably due to a geometric constraint associated with DNA binding through the SRA domain (60,69). In support of this notion, on average both highly methylated low- and high-density CpGs are primarily located in linker DNA (valleys) (Supplementary Figure S6C), and low-density CpG methylation in the baseline methylome is enriched in linker DNA (Supplementary Figure S6D, left) while high-density CpG methylation is enriched across both linker DNA and nucleosome core particles (peaks) (Supplementary Figure S6D, right), an observation consistent with previous analysis of nucleosome occupancy and CpG density (86). Considering these data with genome-level analyses presented in this paper, we hypothesize that CpG density controls the substrate specificity of UHRF1, and that H3 in genomic regions of high CpG density are poor substrates for UHRF1 ubiquitin ligase activity.

### DNMT1 and the role of ubiquitin signaling for DNA methylation maintenance

Differences in recovery dynamics following UHRF1 and DNMT1 knockdown were surprising to us. While DNMT1 re-introduction essentially restored DNA methylation patterns to the pre-knockdown state, UHRF1 re-introduction showed inefficient recovery of low-density CpG methylation in late replicating regions of the genome. This imbalance in low-density CpG methylation mirrors patterns of DNA hypomethylation reported in primary human cancers. Our data supports a model in which UHRF1-dependent H3 ubiquitination ‘bookmarks’ genomic regions of low CpG density in late replicating chromatin for re-methylation by DNMT1, and that a disrupted UHRF1-DNMT1 ubiquitin signaling axis contributes to DNA hypomethylation in cancer. This hypothesis is consistent with the model that links PMD formation in cancer to mitotic divisions (11,15), where competition between the pace of DNA replication and cell division leads to incomplete maintenance DNA methylation. A requirement for signaling through a multi-step write-read-write mechanism of ubiquitin-dependent DNA methylation maintenance may be error prone and unresolvable late in the replication cycle.

Also surprising to us was the observation that ubiquitin reading activity, while dispensable for DNMT1 activity *in vitro*, is required to support the full extent of DNMT1-dependent DNA methylation maintenance in cells. DNMT1 adopts an autoinhibitory conformation when the RFTS (containing its UIMs) sits within the catalytic core (33,34,87–90). Interaction with multi-monoubiquitinated H3 partially releases the RFTS (N-lobe, UIM1) from the catalytic pocket and enhances the methyltransferase activity of DNMT1 (34,39,90). Integrating this observation with our results, we suggest that ubiquitin signaling provides two primary functions for DNMT1-mediated maintenance methylation – partial relief from an autoinhibited state and recruitment of DNMT1 to replicating chromatin. As disruptions to UHRF1 and its ubiquitin ligase activity only partially affect DNMT1-dependent DNA methylation, our data also suggests that other ubiquitin ligases, functioning in a compensatory or independent manner with UHRF1, contribute to DNMT1 maintenance methylation activity. Indeed, NEDD4, CUL4A and PHF7 all have reported overlapping H3 ubiquitin substrate specificity with UHRF1 (91–95).

Finally, we report for the first time distinct functions of the two DNMT1 UIMs, both in binding ubiquitinated nucleosomes and in DNMT1 maintenance methylation activity. The convergent phenotypes of disrupting UHRF1 ubiquitin ligase activity and DNMT1 UIM1 ubiquitin reading activity suggest that DNMT1 UIM1 reads the ubiquitin ‘bookmark’ laid down by UHRF1 to maintain DNA methylation in regions of low CpG density. Loss of DNMT1 UIM2 reading activity, on the other hand, disrupted DNMT1 activity almost to the same extent as catalytic inactivation of the enzyme, suggesting that DNMT1 UIM2 supports DNA methylation maintenance genome-wide. DNMT1 UIM2 may function in concert with other ubiquitin ligases that target the H3 tail, and it may also have critical roles in maintenance methylation beyond the recognition of ubiquitin on the H3 tail.

### Concluding remarks

In summary, we have demonstrated that the ubiquitin ligase activity of UHRF1 is essential for supporting DNMT1-mediated maintenance methylation of low-density CpGs. Additionally, we have provided evidence that disruption to the UHRF1-DNMT1 ubiquitin signaling axis promotes low-density CpG hypomethylation patterning reminiscent of PMD formation observed in ageing and cancer. Finally, we have shown that DNMT1 requires interaction with ubiquitin to mediate DNA methylation maintenance. Collectively, these results demonstrate the complexity of maintaining DNA methylation patterns in dividing cells and provide crucial insight for future work aimed at understanding these mechanisms.

## Supplementary Material

gkae1105_Supplemental_File

## Data Availability

All WGBS, ChIP-seq and EPIC array raw and processed data is deposited in the Gene Expression Omnibus archive under accession GSE256033 (SuperSeries) and will be released to the public upon publication. All code used for processing, analysis, and figure generation is deposited on our GitHub site (https://github.com/rleetied/2024_Tiedemann_et_al_UHRF1_DNMT1) and Zenodo at https://doi.org/10.5281/zenodo.13983528.
